# Beta- Lactam Antibiotics Stimulate Biofilm Formation in Non-Typeable *Haemophilus influenzae* by Up-Regulating Carbohydrate Metabolism

**DOI:** 10.1371/journal.pone.0099204

**Published:** 2014-07-09

**Authors:** Siva Wu, Xiaojin Li, Manjula Gunawardana, Kathleen Maguire, Debbie Guerrero-Given, Christoph Schaudinn, Charles Wang, Marc M. Baum, Paul Webster

**Affiliations:** 1 Life Sciences Division, University of California, Berkeley, California, United States of America; 2 Molecular Diagnostic Laboratory, ApolloGen Inc., Irvine, California, United States of America; 3 Oak Crest Institute of Science, Pasadena, California, United States of America; 4 University of California San Diego, San Diego, California, United States of America; 5 Max Planck Florida Institute, Jupiter, Florida, United States of America; 6 Robert Koch Institute, Berlin, Germany; 7 Center for Genomics and Division of Microbiology and Molecular Genetics, School of Medicine, Loma Linda University, Loma Linda, California, United States of America; 8 Center for Electron Microscopy and Microanalysis, University of Southern California, Los Angeles, California, United States of America; Ghent University, Belgium

## Abstract

Non-typeable *Haemophilus influenzae* (NTHi) is a common acute otitis media pathogen, with an incidence that is increased by previous antibiotic treatment. NTHi is also an emerging causative agent of other chronic infections in humans, some linked to morbidity, and all of which impose substantial treatment costs. In this study we explore the possibility that antibiotic exposure may stimulate biofilm formation by NTHi bacteria. We discovered that sub-inhibitory concentrations of beta-lactam antibiotic (i.e., amounts that partially inhibit bacterial growth) stimulated the biofilm-forming ability of NTHi strains, an effect that was strain and antibiotic dependent. When exposed to sub-inhibitory concentrations of beta-lactam antibiotics NTHi strains produced tightly packed biofilms with decreased numbers of culturable bacteria but increased biomass. The ratio of protein per unit weight of biofilm decreased as a result of antibiotic exposure. Antibiotic-stimulated biofilms had altered ultrastructure, and genes involved in glycogen production and transporter function were up regulated in response to antibiotic exposure. Down-regulated genes were linked to multiple metabolic processes but not those involved in stress response. Antibiotic-stimulated biofilm bacteria were more resistant to a lethal dose (10 µg/mL) of cefuroxime. Our results suggest that beta-lactam antibiotic exposure may act as a signaling molecule that promotes transformation into the biofilm phenotype. Loss of viable bacteria, increase in biofilm biomass and decreased protein production coupled with a concomitant up-regulation of genes involved with glycogen production might result in a biofilm of sessile, metabolically inactive bacteria sustained by stored glycogen. These biofilms may protect surviving bacteria from subsequent antibiotic challenges, and act as a reservoir of viable bacteria once antibiotic exposure has ended.

## Introduction

Non-typeable *Haemophilus influenzae* (NTHi), a Gram-negative, rod-shaped bacterium is a common commensal organism in the human upper respiratory tract. However, NTHi is also an emerging causative agent of chronic infections in humans [1]–[3], imposing a substantial economic burden on health care systems. For example, NTHi is a major causative agent of acute otitis media (AOM, middle ear infection) [4]–[6]. Treatment costs for otitis media in 2006 were estimated to be $2.8 billion per year [7]. Tympanostomy tube insertion, a common treatment for chronic serous otitis media [8], [9], has a treatment cost of $2,174 per patient [10]. NTHi has also been linked to community-acquired pneumonia in children [11], infections in cystic fibrosis patients [12], and upper respiratory tract infections [13]–[17]. NTHi can cause sinus infections [3], [18] and has been linked to morbidity in patients with chronic obstructive pulmonary disease (COPD) [14]–[16].

Orally administered beta-lactam antibiotics are the usual treatment for NTHi infections. For otitis media infections beta-lactam antibiotics such as amoxicillin, ampicillin or cefuroxime are used [19], [20]. Although antibiotic treatments for otitis media are generally successful [19], [20], antibiotic resistance [21], and the ability of otitis media bacteria to form antibiotic tolerant biofilms [6] highlights two major difficulties in treating this disease; antibiotic resistance and increased susceptibility to re-infection after treatment. The emergence of antibiotic resistant pathogens in otitis media has prompted recommendations aimed at limiting antibiotic exposure in children [22]. The reported possibility of increased risk of recurrent otitis media after antibiotic treatment [23] also argue for judicious use of antibiotics in children, as do the links between antibiotic exposure in early life and increased risk of subsequent Crohn’s disease [24] or asthma [25]–[27]. Previous antibiotic treatment is also a risk factor for increased occurrence of acute otitis media caused by NTHi [5], presumably as a result of antibiotic-induced selection or biofilm formation.

Recent studies suggest that antibiotics may promote biofilm formation. For example, a broad range of antibiotics can stimulate *in vitro* biofilm formation by *Staphylococcus epidermidis* [28]–[34]. Similarly, *Pseudomonas aeruginosa* biofilm formation is induced by sub-inhibitory concentrations of tobramycin, tetracycline and norfloxacin [35], [36]. In both organisms, biofilm formation in the presence of sub-inhibitory concentrations of antibiotic is a consequence of altered gene expression. The modulation of bacterial gene expression by antibiotics is a well-documented phenomenon that is usually linked to modification of ribosome function [35], [37], [38]. Although we do not yet know how beta-lactams might modulate gene expression in NTHi bacteria, a subset of beta-lactams have been reported to induce colanic acid production in *Escherichia coli* [39]. In *E. coli*, colanic acid is important for maturation of biofilm architecture, so any increase in its synthesis might exacerbate the formation or persistence of biofilms [39].

Recognition of the role of biofilm infections in otitis media [6] has improved our understanding of why chronic and persistent infections occur, but offers little guidance for successful treatment. The difficulties of treating biofilm infections, which can be up to 1,000 times more resistant to antibiotics [40], has prompted some physicians to propose a gradual move away from traditional antibiotic treatments and toward non-antibiotic therapies [41]. If antibiotics are to continue to be relevant for treating bacterial infections it is important that their effects on biofilms be explored. One step in this direction would be to develop routine screening methods to test the effects of antibiotics on *in vitro* formed biofilms.

In this study we have used a biofilm assay model to explore the effects of exposing newly forming NTHi biofilms *in vitro* to beta-lactam antibiotics. We speculated that the increased risk of NTHi AOM observed after recent antibiotic treatment [5], [23] may be a result of antibiotic-stimulated biofilm formation. We tested this hypothesis *in vitro* by exposing NTHi bacteria to increasing concentrations of beta lactam antibiotic during biofilm formation. We evaluated the formed biofilms using a classical crystal violet biofilm assay and compared the results obtained from biofilms formed in the absence of antibiotic. We also tested the ability of biofilm bacteria previously exposed to beta-lactam antibiotic to resist a subsequent challenge from a lethal dose of cefuroxime. We obtained more accurate measurements of biofilm biomass, protein content and viable bacteria counts for comparisons and have examined the biofilms formed by NTHi bacteria in the presence of beta-lactam antibiotics microscopically. Finally we performed an analysis of mRNA obtained from biofilm bacteria to determine if exposure to beta-lactam antibiotics was able to modulate gene expression in NTHi bacteria.

## Methods

### Bacterial biofilm cultivation

50 µl from frozen stock cultures of non-typeable *Haemophilus influenzae* strains PittAA, PittEE, PittGG, PittII [42], [43], 2019 [44] and 9274 [45] were spread onto chocolate agar plates (Hardy Diagnostics, Santa Maria, CA, USA) and incubated at 37°C in an atmosphere of 5% CO_2_ with 95% relative humidity. After 24-hour incubation, multiple colonies were used to inoculate BHI broth (Bacto Brain Heart Infusion; BD Diagnostics, Sparks, MD, USA) supplemented with hemin (Sigma-Aldrich Inc., St. Louis, MO, USA) at 10 µg/ml and nicotinamide adenine dinucleotide (NAD) (Sigma-Aldrich Inc., St. Louis, MO, USA) at 2 µg/ml (sBHI) [46]. The bacterial suspension was grown to stationary phase (1×10^9^ CFU/ml) at 37°C, 5% CO_2_, with 95% relative humidity. Biofilms in this study were prepared by diluting 1∶200 bacterial suspensions at stationary phase with fresh sBHI, aliquoting into culture vessels appropriate for subsequent applications, and incubating at 37°C with 5% CO_2_ and 95% relative humidity for 24 hours. All NTHi strains were isolated from chronic otitis media patients, with the exception of 2019, which was isolated from a patient with chronic bronchitis.

### Antibiotics

Stock solutions of ampicillin, amoxicillin, and cefuroxime (all from Sigma-Aldrich Inc., St. Louis, MO, USA) were prepared as follows: ampicillin trihydrate and amoxicillin were dissolved in pure DMSO, sterilized using a 0.22 µm syringe filter with nylon membrane (Acrodisc, Pall Corporation, Port Washington, NY, USA), and aliquoted into sterile cryovials for storage at −20°C. Cefuroxime sodium salt was dissolved in BHI broth and sterilized as above. All antibiotic stock solutions were frozen and thawed once before use. All were used immediately after thawing and then discarded.

### Antibiotic susceptibility test

All NTHi strains in this study were tested for susceptibilities to ampicillin and cefuroxime, using the Bauer-Kirby disk diffusion method [47]. Following standard testing procedures and results interpretive charts provided by the manufacturer, BBL Sensi-Discs (BD Diagnostics, Sparks, MD, USA) impregnated with 10 µg ampicillin or 30 µg cefuroxime were applied to growing bacteria prepared using direct colony suspension method, with *H. influenzae* ATCC 49247 and ATCC 49766 (American Type Culture Collection, Manassas, VA, USA) as control strains for sensitivity to ampicillin and cefuroxime.

Minimum inhibitory concentrations (MICs) and minimum bactericidal concentrations (MBCs) were determined on experimental strains by applying BSAC Working Party Report guidelines [48].

### Antibiotic assays

Biofilms were prepared in 96-well tissue culture-treated polystyrene microplates (BD Biosciences, Bedford, MA, USA) with beta-lactam antibiotics at designated concentration ranges. One row of 8 wells was used for each experiment and each well was read individually. Control rows of wells contained sBHI only or bacteria in sBHI with no antibiotics. For ampicillin and amoxicillin assays, additional control wells of bacteria in sBHI containing corresponding dilutions of DMSO were also set up. The bacteria were incubated at 37°C in an atmosphere of 5% CO2 and 95% relative humidity for 24 hours before biofilm quantification using crystal violet assay.

In order to investigate the ability of NTHi biofilms to resist lethal doses of beta-lactam antibiotic, biofilms were formed in the wells of 96 well plates (8 wells per experiment) in the presence or absence of specific sub-inhibitory concentrations of selected beta-lactam antibiotics. The formed biofilms were washed with sterile sBHI and incubated overnight in sBHI containing 10 µg/mL cefuroxime. The biofilms were scraped, agitated to disrupt the biofilm and re-suspend the bacteria, and the numbers of viable bacteria estimated using the drop plate counting method [49].

### Crystal violet biofilm assay

The OD_600_ of the fully hydrated biofilm-containing wells was measured using a SpectraMax M5 microplate reader (Molecular Devices, LLC, Sunnyvale, CA, USA). A crystal violet assay was then applied to quantify biofilm formation [50]–[52]. 1% aqueous solution of crystal violet (Sigma-Aldrich Inc., St. Louis, MO, USA) was added to the biofilm at 20 µl per well, left for 15 min. and then washed. After the stained wells were air-dried, DMSO was added. The OD_600_ of crystal violet in DMSO solution (absorption maxima = 600 nm) was measured in a microplate reader.

### Biofilm analysis

In order to obtain sufficient material for estimating biofilm biomass (dry weight) and protein content, NTHi biofilms were formed in 20×100 mm tissue culture-treated polystyrene dishes (BD Biosciences, Bedford, MA, USA) in the presence and absence of 170 ng/mL ampicillin (3 replicas each). Antibiotics were present during biofilm formation. The culture medium was washed from the formed biofilms with PBS and the biofilm material was freeze-dried under vacuum. Dry weights were measured from batches of pooled biofilms (3 dishes each batch). Protein content was estimated using the BCA Protein Assay Kit (Thermo Scientific Pierce) on biofilm material scraped from dishes and tabulated as percent protein content per biofilm. For CFU counts, biofilms were not dried but were scraped and suspended in sBHI, serially diluted and estimates of total viable bacteria calculated after 24 hr incubation [49]. The results were tabulated as numbers of viable bacteria per mg dry biofilm.

### Statistical Analysis

Data from experimental and control conditions were analyzed using GraphPad Prism (version 6.02, GraphPad Software, Inc., La Jolla, CA). Groups were compared using an unpaired t test with Welch’s correction. Statistical significance was defined as P<0.05.

### Confocal laser scanning microscopy

NTHi biofilms were prepared in 96-well tissue culture-treated imaging microplates (BD Biosciences, Bedford, MA, USA). Fluorescent dyes from FilmTracer Live/Dead Biofilm Viability kit (Molecular Probes, Inc., Eugene, OR, USA) were applied to biofilms at the final concentrations of 5 µM and 30 µM, of Syto9 and propidium iodide, respectively. Biofilms were incubated with the dyes at room temperature for 20–40 minutes before being imaged by LSM710 Meta NLO cLSM (Carl Zeiss Inc, Thornwood, NY, USA). All cLSM images were collected within a window of 40–90 minutes after application of the fluorescent dyes.

### Scanning electron microscopy

For better specimen handling for processing and examination in an SEM, plastic coverslips were used as growth substrate for NTHi biofilms; hence, biofilms were prepared in 20×100 mm tissue culture-treated polystyrene dishes (BD Biosciences, Bedford, MA, USA) with Thermanox plastic coverslips (Nalgene Nunc International, Rochester, NY, USA) placed at the bottom. Then, as previously described [22], biofilms on coverslips were fixed by plunge-freezing in liquid propane, freeze-substituted with ethanol, gradually warmed to 4°C, and critical point dried. The dried biofilms were mounted on a specimen stub, sputter coated in argon with a 18 nm-layer of platinum and examined in the XL 30 SFEG SEM operating at 5 kV in the secondary electron mode (FEI Company, Hillsboro, OR, USA). Biofilm thickness estimates were obtained from regions where the biofilm profile was visible.

### Transmission electron microscopy

Specimen holders for the EMPact2 high-pressure freezer (Leica Microsystems IN, Deerfield, IL) were autoclaved and immersed in BHI medium containing NTHi bacteria (PittGG strain) in the presence or absence of 170 ng/mL ampicillin and incubated overnight at 37°C. The specimen holders were removed from culture, dried on the base and frozen under high pressure. Biofilm present in the specimen holders were dehydrated while still frozen [53] and embedded in Lowicryl HM20 resin under UV light while being maintained at −50°C in an AFS 2 (Leica Microsystems IN, Deerfield, IL). Embedded biofilms were re-orientated for optimal sectioning, re-embedded in epoxy resin and sectioned. Thin sections were mounted on Formvar-coated copper and nickel specimen grids and contrasted with aqueous solutions of uranyl acetate and lead citrate.

Glycogen staining was carried out on thin sections before heavy metal contrasting using a previously described protocol [54]. Sections were treated with 1% aqueous periodic acid for 30 min, washed and then treated with sodium chlorite prepared in acetic acid and water as previously described [54]. Periodic acid exposure was omitted in control experiments [54], [55].

Sections were examined with a Tecnai G2 20 transmission electron microscope (FEI, Hillsboro, OR) and images collected digitally. Images were compiled into figures using PhotoShop. Image modifications consisted of brightness and contrast adjustments applied to the whole image. Image cropping was performed for presentation purposes.

### Glycogen Assay

Replicate preparations (4 replicates) of NTHi biofilms were prepared in Petri dishes using strain Pitt GG in the presence and absence of 170 ng/mL ampicillin. After 24 hr incubation the formed biofilms were washed with PBS, scraped from the plastic substrate, collected in tubes and dried. The dried biofilms were weighed and a commercially available glycogen assay kit (Abcam cat # ab65620: www.abcam.com/Glycogen-Assay-Kit-ab65620.html) was used to estimate glycogen content following the protocols supplied with the kit.

### Gene expression transcriptomic profiling by DNA microarray

NTHi PittGG bacteria were suspended in sBHI and added to 20×100 mm tissue culture-treated polystyrene dishes as described above and incubated for 24 hr at 37°C. Three biological replicates per condition were prepared (plus or minus 170 ng/mL ampicillin) and the experiment was carried out as follows.

Briefly, the biofilms were gently washed in PBS to remove culture medium and then removed from the plastic substrate by scraping in 4 mL of PBS. The scraped biofilm suspensions were placed in 50 mL Falcon tubes, evenly dispersed and aliquoted for further processing. The aliquots were centrifuged at 8,000 rpm in a benchtop centrifuge (Eppendorf, Hauppauge, NY) for 3 min. and the supernatant was discarded. The pellets were re-suspended in RNAwiz and total RNA was extracted using Ambion RiboPure-Bacteria kit following the manufacturer’s instructions (Life Technologies Corporation, Carlsbad, CA, USA).

cDNA was synthesized using Invitrogen SuperScript Double-Stranded cDNA Synthesis Kit (Life Technologies Corporation, Carlsbad, CA, USA). Both RNA and cDNA quality was checked using Agilent 2100 Bioanalyzer and Agilent RNA 6000 nano and DNA1000 chips (Agilent Technologies, Inc., Santa Clara, CA, USA). cDNA (0.5–1 µg) was used to start the amplification and labeling reaction using the Roche NimbleGen One-Color DNA Labeling kit, in which Cy3 was randomly incorporated in the newly synthesized DNA by *Klenow* fragment (3’→5’ exo-).

Two µg of labeled DNA derived from each RNA sample was hybridized to each array for over 16–18 hours. DNA microarray gene expression was carried out using Roche NimbleGen custom whole genome tilling arrays (100910_CW_P_ging_W83_expr_HX12) (Roche NimbleGen, Inc., Madison, WI, USA) according to the standard NimbleGen procedure (NimbleGen Arrays User’s Guide: Gene Expression Analysis v5.1).

The slides/arrays were washed and spun dry and then scanned using a NimbleGen MS200 Microarray scanner with a resolution of 2 µm. The normalization was done using NimbleScan 2.6.0.0 built-in normalization function. Microarray data analysis was performed using Partek Genomics Suite v6.5 (Partek Inc., St. Louis, MO, USA). Differentially expressed genes were determined using fold-change (≥1.5) plus P (≤0.05, T-test) with FDR≤0.05. The gene expression data was deposited in NCBI's Gene Expression Omnibus (http://www.ncbi.nlm.nih.gov/geo).

## Results

### Biofilm formation is stimulated by beta-lactam antibiotics

**Figure 1:**
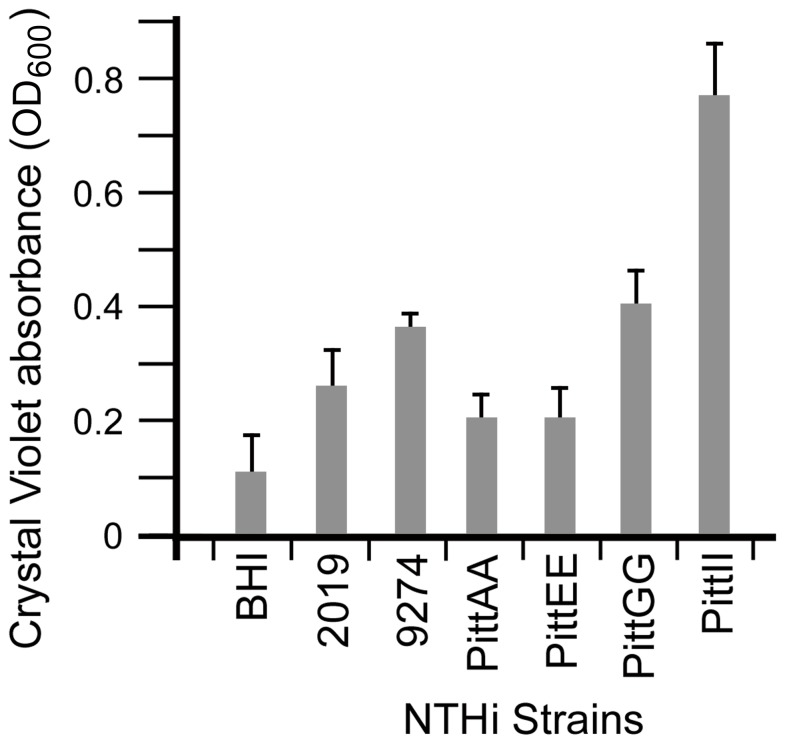
Biofilm formation is variable between NTHi strains. Biofilm quantification using crystal violet assay showed the variability in biofilm formation between the NTHi strains in this study. Relative Biofilm Quantity was measured as OD_600_ of crystal violet in DMSO; higher absorbance indicated more biofilm production. The PittAA and PittEE strains showed the least amount of biofilm attachment to the growth surface while PittII showed the most. The other three strains (2019, 9274 and PittGG) formed intermediate amounts of biofilm material (t-test analysis in Table S2).

A total of six strains of NTHi bacteria were chosen for this study based on their origin and history. All were clinical strains but four had only a limited history of laboratory culture (PittAA, PittEE, PittII, PittGG). The two other strains, 2019 and 9274 are considered to be laboratory strains. Crystal violet staining showed that all the NTHi strains used in this study formed biofilms, as all resulted in OD_600_ readings greater than the sBHI control (Fig. 1 and Table S1). The amount of biofilm formation was variable between strains, reflecting the relative amount of stainable biofilm material attaching to the surface of 96 well plates. NTHi strain PittII produced the most biofilm (Fig. 1) while PittAA and PittEE produced the least (Fig. 1).

Antibiotic resistant varied between the strains as tested using Sensi-Disc test discs (Table S2). Control strains 49249 and 49766 were resistant or susceptible respectively. Most strains were susceptible to cefuroxime with PittEE showing an intermediate reaction. Pitt AA, PittEE and PittGG were resistant to 10 µg ampicillin. However, MIC’s and MBC’s showed that PittAA and PittGG were susceptible to lower amounts of ampicillin than expected. PittAA, resistant to ampicillin as indicated by the test disc assay was only sensitive to high levels of ampicillin (MIC = 800 µg/mL; MBC 1,000 µg/mL). MIC and MBC data is tabulated in Table S2.

**Figure 2:**
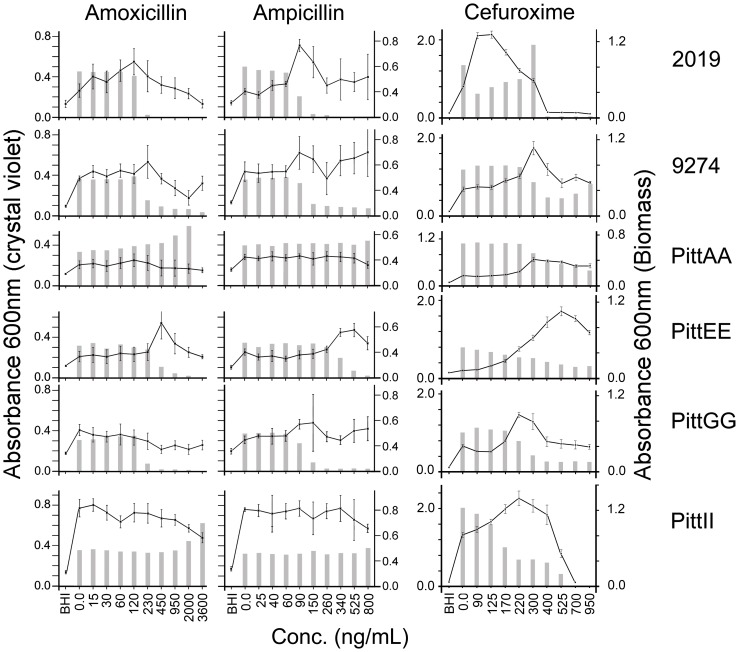
Biofilm formation is stimulated by beta-lactam antibiotics. Crystal violet assays of 1-day NTHi biofilms formed in the presence of amoxicillin or amoxicillin. Strains 2019, 9274 and PittEE reacted to inhibitory concentrations of antibiotic (grey vertical bars, Biomass OD_600_) by producing more crystal violet stainable biofilm (line graph). This stimulatory effect was different for each bacterial strain. PittGG did not react with amoxicillin and produced an ambiguous reaction to ampicillin. PittAA and PittII were not affected by amoxicillin or ampicillin in the concentration ranges studied. Cefuroxime (0–950 ng/mL range) showed a biofilm-stimulatory effect on all the NTHi strains under study. Each strain exhibited biofilm stimulation in different concentrations of antibiotic. Strain 2019 was maximally stimulated at 100 ng/mL cefuroxime, PittGG and PittII at 220 ng/mL, 9274, PittAA at 300 ng/mL and PittEE at 525 ng/mL.

We examined the ability of these NTHi strains to form biofilms in the presence of increasing concentrations of penicillin-based beta-lactam antibiotics. Some of the NTHi strains reacted to the antibiotics by producing more biofilm (Fig. 2). Our experiments showed this stimulatory effect on NTHi strain 2019 in the presence 90 ng/mL ampicillin (*p* = <0.0001) but not in the presence of ampicillin (Fig. 2 and Table S1). Biofilm production was stimulated in 9274 by 230 ng/mL amoxicillin and 90 ng/mL ampicillin (p = 0.002 & 0.0095 respectively). PittAA was stimulated by 120 ng/mL amoxicillin (p = 0.0003);, PittEE by amoxicillin at 450 ng/mL (*p* = <0.0001) and ampicillin at 525 ng/mL (*p* = <0.0001). PittGG and PittII did not appear to react to the presence of amoxicillin or ampicillin in our chosen concentration range, even though there were significant differences between the effects of maximal observed stimulation with unexposed biofilm levels (Fig. 2 and Table S1). PittGG biofilms reached peaks in crystal violet assays in the presence of 60 ng/mL amoxicillin (*p* = 0.0047) and 170 ng/mL ampicillin (*p* = 0.0336). PittII reacted to 120 ng/mL amoxicillin (*p* = <0.0001). However, the stimulated levels of PittGG and PittII in response to amoxicillin were higher than other biofilms formed in the presence of antibiotic but were lower than the untreated biofilms.

Cefuroxime, a cephalosporin beta-lactam antibiotic produced a biofilm stimulatory effect in the range of 0–950 ng/mL on all of the NTHi strains we studied (p = <0.0001) (Fig. 2 and Table S1). Biofilm stimulation occurred at different concentrations depending on the NTHi strain. PittGG and PittII showed maximum biofilm stimulation at 220 ng/mL cefuroxime, PittEE at 525 ng/mL, PittAA and 9274 at 300 ng/mL, and 2019 at 100 ng/mL (Fig. 2). The strongest and broadest reaction was demonstrated by strain PittII, where the biofilm stimulatory effect was observed from 170 ng/mL to 400 ng/mL. The NTHi strain least affected by cefuroxime was PittAA, which showed only a small increase in biofilm formation at 300 ng/mL (Fig. 2 and Table S1).

**Figure 3:**
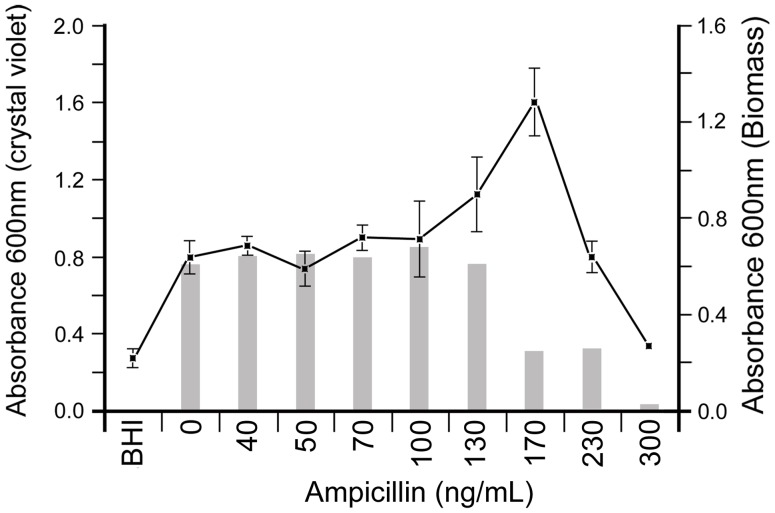
PittGG biofilm formation is stimulated by ampicillin in a narrow concentration range. Crystal violet assay of 1-day PittGG biofilms, formed in the presence of increasing amounts of ampicillin up 300 ng/mL, showed a stimulation of biofilm formation at 170 ng/mL concentration.

The results we obtained for PittGG using the biofilm assay showed no apparent reaction in the 150 ng/mL dilution of ampicillin (Fig. 2). The high SD and low p value (0.0336) prompted us to re-examine the interaction of this strain with a narrower concentration range of ampicillin. Once we narrowed the range in which the antibiotic was tested, we were able to detect a conclusive biofilm-stimulatory effect by ampicillin at a concentration that peaked at 170 ng/mL (*p* = <0.0001) (Fig. 3 and Table S1).

### Beta-lactam antibiotics inhibit bacterial growth but increase biomass and protein content of *in vitro* formed biofilms

**Figure 4:**
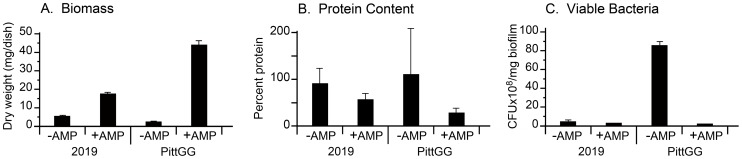
A sub-inhibitory concentration of ampicillin changes the composition of forming NTHi biofilms. Two strains of NTHi, 2019 and PittGG, exposed to sub-inhibitory concentrations (90 ng/mL: 2019; 170 ng/mL: PittGG) of ampicillin for 24 hr, showed increases in biofilm biomass as measured by dry weight (A. Biomass), and percent protein content (B. Protein Content). In contrast, the presence of the antibiotic during biofilm formation resulted in a decrease in viable (or culturable) bacteria (C. Viable Bacteria). Strain PittGG showed the most noticeable changes in dry weight, protein content and numbers of viable bacteria resulting from exposure to 170 ng/mL ampicillin (t-test *p* = <0.05).

In order to determine how beta-lactam antibiotics were affecting the biofilm-forming abilities of NTHi bacteria we assayed the following; a) total biomass of the formed biofilms; b) protein content; and c) total number of viable bacteria in the biofilms. These assays were applied to biofilms formed in the presence and absence of sub-inhibitory concentrations of beta-lactam antibiotics and the results presented in Figure 4.

Results from NTHi strain 2019 showed that in the presence of a sub-inhibitory concentration of ampicillin (94 ng/mL) the NTHi bacteria produced an increased amount of biofilm biomass (dry weight in mg per total biofilm per dish). The dry weight increased from 5.3 mg/dish to 17.3 mg/dish (Fig. 4A). Ampicillin applied to NTHi strain PittGG under similar conditions, but using a different maximum stimulatory concentration (161 ng/mL) of antibiotic, produced similar trends in the biofilm biomass (Fig. 4). The biomass of the untreated PittGG biofilm was 2 mg/biofilm, increasing to 44 mg/biofilm when formed in the presence of ampicillin (Fig. 4A)

The presence of ampicillin resulted in a reduction in the relative protein composition in the 2019 and PittGG biofilms (Fig. 4B). While most of the untreated biofilm was composed of protein (2019: 91%, PittGG: over 100%), ampicillin-treated biofilms contained less protein (2019: 57%, PittGG: 28%, Fig. 4B). However, as the total biomass of the biofilm had increased in the presence of the antibiotic, the total amount of protein detected in the ampicillin-treated biofilms was more than double that of the untreated biofilms (for 2019: 4.8 mg/untreated biofilm v. 9.9 mg/treated biofilm: for PittGG: 2.2 mg/untreated biofilm v. 12.1 mg/treated biofilm, Fig. 4). The anomalous measurement obtained from the untreated PittGG biofilm (over 100% protein content) was most probably due to the inability of the protein assay to detect the low level of protein present in the biofilm (approx. 2.2 mg/biofilm). This possibility is supported by the large coefficient of error present in the readings. Additional measurements did not produce more accurate results (data not shown).

The presence of ampicillin reduced the numbers of viable bacteria (in CFU’s per mg biofilm) in forming biofilms (Fig. 4C). The numbers of viable bacteria in the 2019 biofilms decreased from 4.1×10^8^ (no ampicillin) to 2.4×10^8^ (with ampicillin). Viable bacteria in the PittGG biofilms decreased from 8.5×10^9^ (no antibiotic) to 1.8×10^8^ (with antibiotic).

### Antibiotic resistance does not predict a biofilm stimulatory response

Presuming that antibiotic resistance may play a role in the biofilm-stimulatory effect on the NTHi bacteria, we screened all the strains for antibiotic resistance using routine methods (Table S2). PittAA and PittEE strains were completely resistant to ampicillin. All the other strains used in this study did not exhibit resistance to the remaining three antibiotics. Minimum inhibitory concentrations (MICs) and minimum bactericidal concentrations (MBCs) for the NTHi strains occurred over a broad µg/mL range of dilutions, and much higher than the ng/mL concentration range used to promote biofilm formation.

### Biofilm bacteria survive sub-inhibitory concentrations of ampicillin

**Figure 5:**
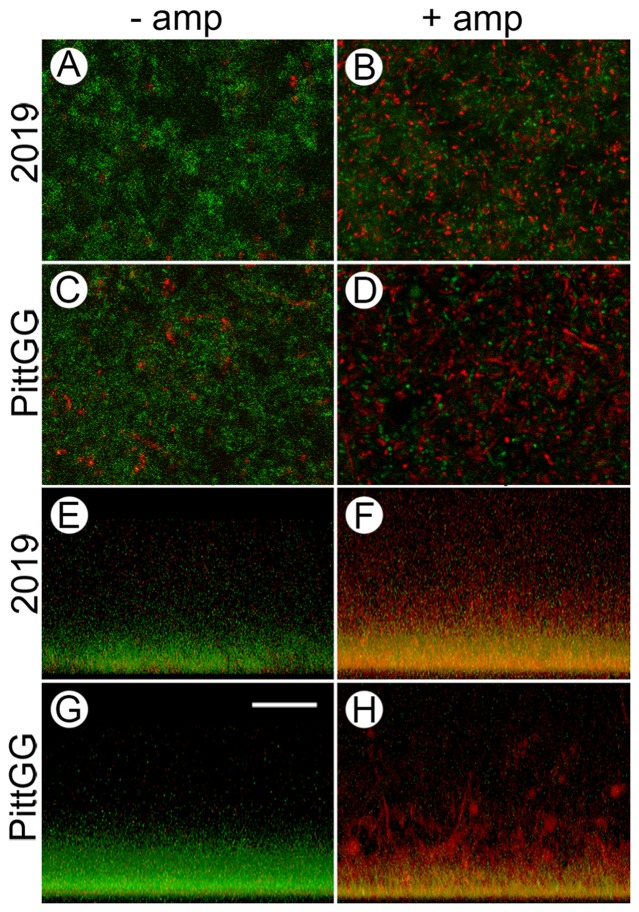
Sub-inhibitory concentrations of ampicillin result in an increase in dead NTHi bacteria in newly formed biofilms. Confocal laser scanning microscopy (cLSM) of biofilms formed by NTHi strains 2019 and PittGG in the absence (− amp) and presence (+ amp) of sub-inhibitory concentrations of ampicillin were stained using the LIVE/DEAD viability assay. 2019 were exposed to 90 ng/mL ampicillin and PittGG to 170 ng/mL ampicillin. Live bacteria were colored green and dead bacteria red. From above, biofilms formed in the absence of ampicillin were mostly green (A & C), indicating living (or intact) NTHi bacteria. Clumps of bacteria in both biofilms stained red (A & C) indicating the presence of dead, or structurally compromised bacteria. Biofilms formed by 2019 (A) contained fewer aggregates of red bacteria than did PittGG (C) contained more. In the presence of ampicillin (B & D) the bacteria in the biofilms were mostly red, indicating a large number of dead bacteria. Green-stained bacteria were still present in both biofilms but appeared more aggregated in the presence of antibiotic (B & D). Large amounts of aggregated red bacteria were present in both biofilms, with the aggregates being larger in the PittGG biofilm (Fig. D). Z-stack projections of the biofilms (E–H) showed that all the biofilms were denser at the base of the biofilm, whether exposed to ampicillin or not. In the absence of ampicillin, 2019 (E) and PittGG (G) biofilms comprised green, or intact bacteria. In the presence of ampicillin the biofilm contained mostly structurally compromised bacterial cells, which were colored red or yellow. The 2019 (F) and PittGG (H) biofilms formed in the presence of ampicillin were higher than the comparable biofilms formed without exposure to ampicillin (E & G). All images; scale bar (G) = 20 µm.

The Live/Dead stain was applied to biofilms formed in the presence and absence of ampicillin. Untreated biofilms of NTHi strains 2019 and PittGG contained mostly green-staining small dots (live bacteria), with only few red (dead) bacteria (Figs. 5A & C). Untreated PittGG biofilms appeared to contain more regions of dead bacteria than the 2019 biofilms (Figs. 5C & D).

The presence of sub-inhibitory concentrations of ampicillin altered the Live/Dead staining patterns in both 2019 and PittGG biofilms (Figs. 5B & D). Ampicillin-treated biofilms were stained mostly red, indicating large numbers of structurally compromised or dead bacteria. However, living (green staining) bacteria were still present in the ampicillin-treated biofilms (Fig. 5).

Reconstructed Z-series stacks through the biofilms showed untreated biofilms composed of mostly intact bacteria, and ampicillin-treated biofilms composed of mostly damaged bacteria (Figs. 5E–F). The reconstructed stacks showed that all the bacteria from both NTHi strains were more densely packed at the base of biofilms even when they were structurally compromised (Figs. 5E–H). The untreated biofilms formed by 2019 (Fig. 5E) were of lower height than those formed by PittGG (Fig. 5G), and although it was difficult to determine where the top of the biofilms were using the cLSM, the antibiotic exposure did appear to increase the biofilm thickness in both NTHi strains (Figs. 5F & H). PittGG biofilms formed in the presence of ampicillin contained large numbers of structurally compromised bacteria in red stained aggregates. The Z-stack reconstruction of the ampicillin-exposed PittGG biofilm revealed clumps of aggregated dead bacteria (Fig. 5H).

### Ampicillin affects the morphology of *in vitro* formed NTHi biofilms

**Figure 6:**
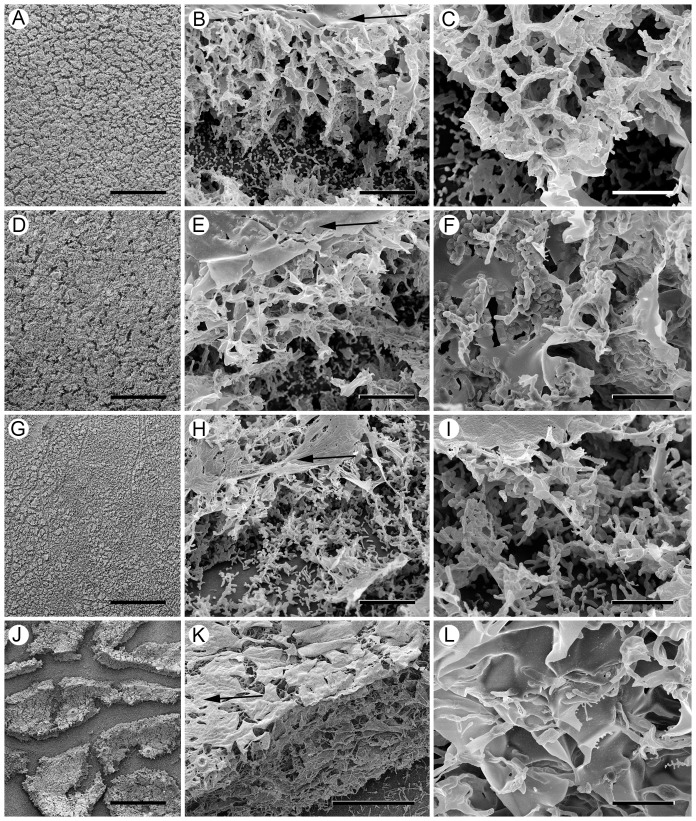
Sub-inhibitory concentrations of ampicillin change the ultrastructure of newly formed NTHi biofilms. Scanning electron microscopy (SEM) images comparing NTHi biofilms formed in the absence and presence of ampicillin on Thermanox coverslips. A–C: SEM of 2019 biofilms. A) At low power the 2019 biofilm is seen as a mat covering the Thermanox substrate. Bar = 500 µm. B) higher magnification shows the biofilm to be composed of partitions forming empty spaces, or cells, covered with a film of amorphous material (arrow). Bar = 10 µm. C) the partitions within the biofilm are composed primarily of bacterial cells aggregated into flat sheets. Bar = 5 µm. D–F: SEM of 2019 biofilms formed in 90 ng/mL ampicillin. D) The 2019 biofilm formed with ampicillin covers the substrate. Bar = 500 µm. E) The biofilm is composed of partitions around empty spaces with a flat sheet of amorphous material (arrow) over the top. Bar = 50 µm F) The biofilm partitions are composed of aggregated bacteria embedded in sheets of amorphous material. Bar = 5 µm. G–I: SEM of PittGG biofilms. G) The PittGG bacteria form a biofilm over the Thermanox surface. Bar = 500 µm. H) The biofilm is composed of bacterial cells aggregated into poorly defined partitions and covered with a layer of amorphous material (arrow). Bar = 10 µm. I) The biofilm is composed of bacterial cells aggregated into widely spaced strands, and empty space. Bar = 5 µm. J–L: SEM of PittGG biofilms formed in 170 ng/mL ampicillin. J) In the presence of ampicillin the PittGG bacteria form a biofilm comprised of a thick mat, which appears to be strongly attached to itself but less well attached to the substrate. Bar = 500 µm. K) The biofilm mats appear to be composed mostly of amorphous material, a layer of which covers the biofilm (arrow), and arranged into tightly-packed thin partitions. Bar = 50 µm. L) the biofilm is composed of amorphous material formed into thin partitions. The few bacteria detected were embedded in the thin partitions. Bar = 5 µm.

In order to examine the NTHi biofilms at higher resolution, biofilms on Thermanox slides formed under sBHI broth were examined by scanning electron microscopy (SEM). At low magnification, the biofilm formed by strain 2019 appeared as a uniformly thick mat covering the substrate (Fig. 6A) with thin breaks running through the mat, which probably formed during specimen preparation. At higher magnification, the biofilm, which could be easily examined between the breaks, was approx. 30 µm high and consisted of a network of partitions and spaces covered with a thin layer of amorphous material (Fig. 6B). The partitions running through the biofilm were composed mostly of aggregated bacterial cells (Fig. 6C).

NTHi strain 2019 biofilms that formed in the presence of sub-inhibitory amounts of ampicillin (Figs. 6D–F) were morphologically similar to the untreated biofilms when examined at low magnification and had breaks running through the mat (Fig. 6D). At higher magnification (Fig. 6E), the ampicillin-treated NTHi 2019 biofilm was approx. 40 µm high and was composed of a network of partitions and spaces covered on the top surface with a thick amorphous layer. However, when examined more closely, the partitions in the biofilm appeared to contain fewer aggregated bacterial cells (Fig. 6F) than in the untreated biofilm (Fig. 6C), and instead were composed of sheets of amorphous material containing embedded bacterial cells (Fig. 6F).

PittGG biofilms formed on Thermanox slides were approx. 20 µm high, and therefore thinner than the NTHi 2019 biofilms (Figs. 6G–I) and had few partitions and spaces. The partitions were covered with an amorphous layer (Fig. 6) and contained fewer aggregated bacteria (Fig. 6I) than observed in the 2019 biofilm (Fig. 6C). Ampicillin exposure produced the most striking morphological changes in the PittGG biofilms. In contrast to biofilms formed in the absence of antibiotic (Fig. 6G), the biofilms formed in the presence of ampicillin were thick mats separated by wide breaks (Fig. 6J). These islands of biofilm were not strongly attached to the substrate and could be easily dislodged during processing. The mats were substantially thicker (approx. 80 µm) than either the untreated PittGG biofilm or the biofilms formed by the 2019 strain in the presence or absence of antibiotic. As with the other biofilms, the mats were covered on the top surface with amorphous material (Fig. 6K) and were composed of partitions and spaces. The partitions within the biofilm were made from thin films of amorphous material. Unlike the other biofilms, few bacterial cells were detected embedded in these partitions (Fig. 6L).

### Amoxicillin-stimulated biofilms protect bacteria from a lethal dose of cefuroxime

**Figure 7:**
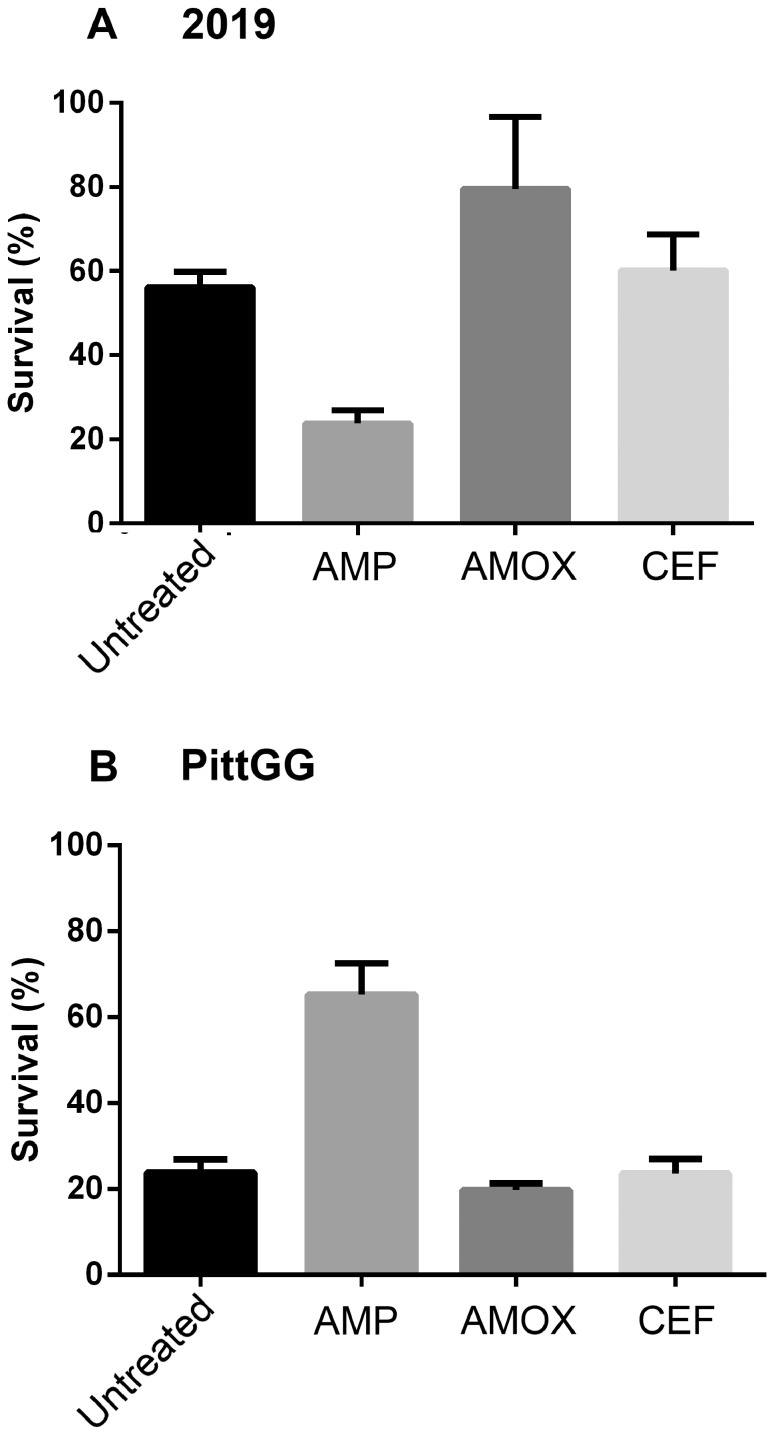
NTHi biofilms protect against a lethal dose of cefuroxime. Biofilms of NTHi strains 2019 and PittGG were formed overnight in the absence of antibiotic (Untreated), or in the presence of 170 ng/mL ampicillin (AMP), 230 ng/mL amoxicillin (AMOX) or 170 ng/mL cefuroxime (CEF). The amounts of antibiotic were chosen for their ability to stimulate biofilm formation in these two NTHi strains. After washing, the biofilms were exposed to 10 µg/mL of cefuroxime for a further 24 hr. The percentages of viable bacteria present in each biofilm are tabulated here. Not shown: planktonic NTHi bacteria are killed in the presence of 10 µg/mL cefuroxime. Although all of the formed biofilms (in the presence or absence of antibiotic) protected against cefuroxime, the amoxicillin-stimulated biofilm was able to protect bacteria from the lethal effects of the cefuroxime. p-values are documented in Table S3.

Suspensions of planktonic forms of NTHi strains 2019 and PittGG showed no signs of bacterial growth after an overnight incubation in 10 µg/mL concentration of cefuroxime (data not shown), indicating bacterial killing by the high dose of antibiotic. However, if the two strains of NTHi were first allowed to form a biofilm and the biofilm bacteria then exposed to the 10 µg/mL dose of cefuroxime, the antibiotic was unable to completely eliminate all viable bacteria from the biofilm (Fig. 7).

Exposure to sub-inhibitory concentrations of beta-lactam antibiotic modified the sensitivity to the subsequent challenge from the high cefuroxime dose. The modification varied depending on the strain of NTHi examined and on the first antibiotic the bacteria encountered (Fig. 7 and Table S1). The 2019 strain showed a significant increased sensitivity to the lethal dose of cefuroxime after an initial exposure to 170 ng/mL ampicillin (*p* = <0.0001) when compared to bacteria from biofilm alone (Fig. 7A). However, exposure to 230 ng/mL amoxicillin produced a decrease in cefuroxime sensitivity (*p* = <0.0059). Exposure to 170 ng/mL of cefuroxime did not change sensitivity to the subsequent lethal dose of cefuroxime (Fig. 7A) and reacted in a similar way to bacteria from untreated biofilms (Fig. 7).

The biofilms formed by the PittGG strain showed a different trend. Exposure to a sub-inhibitory dose of ampicillin significantly decreased the sensitivity of the PittGG bacteria to the high dose of cefuroxime (*p* = <0.0001, Fig. 7B and Table S3). Bacteria from biofilms formed in a sub-inhibitory dose of ampicillin were more sensitive to the high dose of cefuroxime (*p* = <0.0001). An initial sub-inhibitory dose of cefuroxime did not alter sensitivity to the subsequent higher dose (Fig. 7B).

### Ampicillin affects mRNA expression

**Figure 8:**
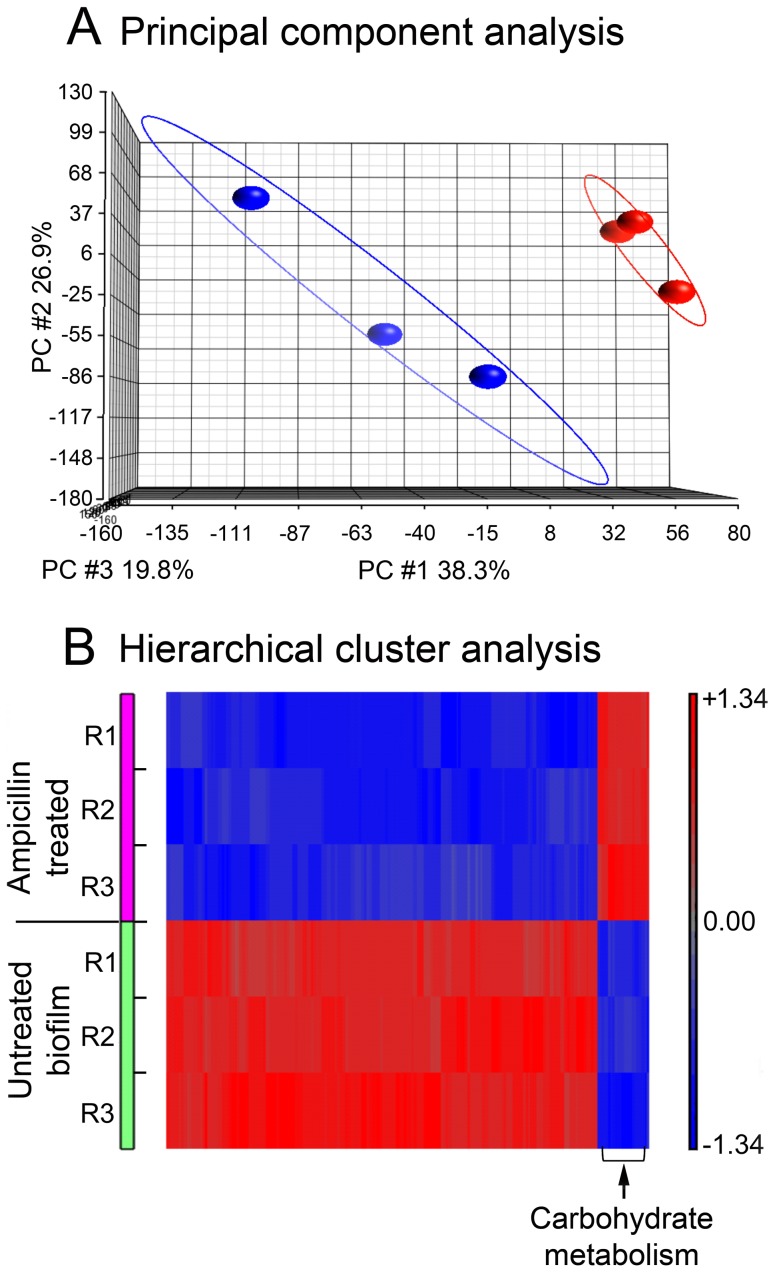
mRNA analysis of PittGG biofilms. A. Principal component analysis of microarray gene expression of the PittGG biofilm bacteria (NTHi) exposed to 170 ng/mL ampicillin. The major variations (PC1, PC2, and PC3) are visualized in 3-dimensions. The three PCs together accounted for about 85% of the variations present in the entire data set. A distinguishable grouping difference can be seen between ampicillin treated (in blue) and non-treated (in red) samples. B. Hierarchical cluster of significantly differentially expressed genes in the PittGG biofilm bacteria (NTHi) treated with 170 ng/mL ampicillin. Heatmap visualization of 59 significantly regulated genes by ampicillin treatment. Results from the 3 replicate experiments (R1, R2 and R3) of ampicillin-treated and untreated biofilms are presented. A hierarchical clustering was analysed on the probe sets representing the 59 genes including 8 up and 51 down regulated gene transcripts with filter criteria of at least 1.5 folds change plus P≤0.05 and FDR less than 0.05. Rows: samples; columns: genes. The up-regulated genes relating to carbohydrate metabolism are indicated.

In order to determine the underlying molecular cause for different biofilm phenotype formation in the presence or absence of a sub-inhibitory dose of ampicillin, we performed microarray gene expression profiling using Roche NimbleGen whole genome custom tilling array (12×135 K) for *H. influenzae* and the RNA from PittGG biofilms formed in the presence and absence of antibiotic. Principal component analysis (PCA) on microarray whole transcriptome expression profiling showed that the PittGG biofilm samples where bacteria (NTHi) were treated with ampicillin were clearly different from the control samples (Figure 8). Significant differentially expressed genes (DEGs) were identified based on fold change (≥1.5-fold) plus p value (≤0.05) with a FDR≤0.05. The microarray assay identified 59 DEGs that met the above criteria in the NTHi bacteria treated with 170 ng/mL ampicillin as compared with the control (Table 1). Eight genes were up-regulated and 51 were down-regulated. Of the 8 up-regulated genes 5 were linked to carbohydrate metabolism (Figure 8), one was involved in pyrimidine metabolism and two were transporters (an ABC transporter and a sodium: hydrogen antiporter). Down-regulated genes were less easily grouped based on their functions because the genes spanned a variety of metabolic processes, including cell wall synthesis (Table 1 and Figure 8). However, some of the most down-regulated genes were also involved with carbohydrate metabolism (Table S4). The gene expression data has been deposited in NCBI's Gene Expression Omnibus and is accessible through GEO Series accession number GSE58213 (http://www.ncbi.nlm.nih.gov/geo/query/acc.cgi?acc=GSE58213).

**Table 1:** Significant genes identified by microarray transcriptomic analysis in the biofilm bacteria exposed to 170/mL ampicillin.

UPREGULATED GENES						
	Gene Name	Ordered Locus	Uniprot	Fold Change	p-Value	Biological Process/function
**Carbohydrate Metabolism**						
ADP-glucose synthase	glgC	NTHI1807	Q4QK69	1.66	0.0008	glycogen biosynthesis
4-alpha-glucanotransferase	malQ	NTHI1810	Q4QK66	1.58	0.0003	glycosyltransferase
1,4-alpha-glucan branching enzyme	glgB	NTHI1809	Q4QK67	1.62	0.0007	glycogen biosynthesis
Glycogen operon protein GlgX	glgX	NTHI1808	Q4QK68	1.55	0.0009	glycogen catabolism
Glycogen synthase	glgA	NTHI1806	Q4QK70	1.86	0.0003	glycogen biosynthesis
**Transporter**						
Predicted cobalt transport protein		NTHI1421	Q4QL56	1.57	0.001	ABC transporter
**Pyrimidine Metabolism**						
Cytidine deaminase	cdd	NTHI1816	Q4QK60	1.65	0.0006	UMP synthesis
**Hypothetical Protein**						
Uncharacterized protein:pH regulation		NTHI0443	Q4QNL4	1.58	0.001	Na:H antiporter activity
**DOWNREGULATED GENES**						
**Amino-acid biosynthesis**						
Bifunctional protein FolD	folD	NTHI0864	Q4QMI7	−2.36	0.001	Amino-acid biosynthesis
Phosphoglycerol transferase-like protein		NTHI1918	Q4QJX3	−2.09	1E-05	Transferase
**Amino Acid Metabolism**						
Anthranilate synthase component I	trpE	NTHI1768	Q4QK98	−1.52	0.001	Lyase
Peptide methionine sulfoxide reductase	msaB	NTHI1677	Q4QKI0	−1.56	0.001	Oxidoreductase
Glutamate racemase	murI	NTHI205	Q4QJK7	−1.77	0.001	Cell wall biogenesis
**Folate Biosynthesis**						
dihydroneopterin aldolase	folB	NTHI0372	Q4QNS3	−1.71	0.001	Lyase
**Purine Metabolism**						
Bifunctional purine biosynthesis protein	purH	NTHI1051	Q4QM21	−1.98	0.001	Hydrolase
Phosphoribosylaminoimidazole carboxylase ATPase subunit	purK	NTHI1424	Q4QL53	−1.96	0.001	Lyase
N5-carboxyaminoimidazole ribonucleotide mutase	purE	NTHI1425	Q4QL52	−3.70	5E-05	Lyase
Phosphoribosylformylglycinamidine cyclo-ligase	purM	NTHI1704	Q4QKF3	−3.45	9E-05	Ligase
Phosphoribosylaminoimidazole-succinocarboxamide synthase	purC	NTHI2033	Q4QJM1	−2.50	0.0003	Ligase
Phosphoribosylglycinamide formyltransferase	purN	NTHI1706	Q4QKF2	−2.66	1E-04	Transferase
adenylosuccinase	purB	NTHI0758	Q4QMS8	−1.52	0.001	Lyase
**Regulation of transcription**						
BirA bifunctional protein	birA	NTHI0323	Q4QNW6	−2.63	0.0004	Ligase
Ribose operon repressor	rbsR	NTHI0634	Q4QN40	−1.62	0.001	DNA binding
Xylose operon regulatory protein	xylR	NTHI1273	Q4QLI5	−2.15	9E-05	DNA binding
**Toxin biosynthetic process**						
Colicin V production protein	cvpA	NTHI1377	Q4QL93	−1.92	0.001	Membrane protein
**Carbohydrate metabolism**						
D,D-heptose 1,7-bisphosphate phosphatase		NTHI0880	Q4QMH3	−3.21	0.0003	Hydrolase
Fructose-1,6-bisphosphatase	glpX	NTHI0789	Q4QMP9	−1.85	0.002	Hydrolase
Acetoacetate CoA transferase alpha subunit	atoD	NTHI0935	Q4QMC5	−1.75	0.0006	Transferase
**DNA replication**						
DNA polymerase III subunit epsilon	dnaQ	NTHI0223	Q4QP50	−2.29	7E-05	Transferase
Ribonuclease H	rnhA	NTHI0224	Q4QP49	−3.09	0.0001	Endonuclease
**Protein transporter**						
Competence protein E	comE	NTHI0560	Q4QNB0	−1.87	0.001	Protein secretion
Type IV pilin secretion protein	pilB	NTHI0408	Q4QNP5	−1.64	0.0006	
Type IV pilin secretion protein	pilC	NTHI0407	Q4QNP6	−1.74	0.001	
**Cellular cell wall organization**						
Membrane-bound lytic murein transglycosylase F	mltF	NTHI0338	Q4QNV4	−2.21	2E-04	
**Aminoacyl-tRNA biosynthesis**						
Lysine–tRNA ligase	genX	NTHI1003	Q4QM64	−1.60	0.001	
**ABC transporter**						
periplasmic oligopeptide-binding protein	oppA	NTHI1292	Q4QLH0	−1.74	0.0003	
ABC-type chelated iron transport system	hfeD	NTHI0477	Q4QNI3	−1.5	0.001	
**DNA packaging**						
Phage terminase large subunit		NTHI1741	Q4QKB9	−2.02	0.001	
**Protein deacetylation**						
NAD-dependent deacetylase sirtuin 5		NTHI1634	Q4QKL5	−2.80	2E-04	Hydrolase
**RNA repair**						
Multifunctional CCA protein	cca	NTHI1436	Q4QL41	−1.97	0.002	
**Amino acid transporter**						
Tryptophan-specific transport protein	mtr	NTHI0396	Q4QNQ4	−1.84	0.0002	
**Hypothetical Proteins**						
		NTHI0215	Q4QP56	−3.28	0.001	transmembrane transport
		NTHI0680	Q4QMZ6	−1.73	0.0004	
		NTHI0735	Q4QMU6	−1.64	0.0005	Predicated membrane protein
		NTHI1330	Q4QLD5	−1.64	0.001	
Conserved FAD/FMN-containing dehydrogenase		NTHI1331	Q4QLD4	−1.77	0.001	
		NTHI1505	Q4QKY1	−2.25	0.001	
		NTHI1511	Q4QKX6	−2.54	0.0007	DNA replication
		NTHI1528	Q4QKW1	−2.17	0.0005	
		NTHI1534	Q4QKV5	−1.84	0.002	
		NTHI1635	Q4QKL4	−4.08	0.001	Catalytic activity
		NTHI1721	Q4QKD8	−3.11	0.001	DNA binding
		NTHI1726	Q4QKD3	−1.92	0.0006	
		NTHI1735	Q4QKC5	−2.31	0.001	
		NTHI1736	Q4QKC4	−2.10	0.0008	Cell wall catabolism
		NTHI1738	Q4QKC2	−2.04	0.0003	
		NTHI1739	Q4QKC1	−2.12	0.0005	DNA binding
Putative recombination protein NinB	ninB	NTHI1727	Q4QKD2	−2.54	0.0006	
Putative recombination protein NinG	ninG	NTHI1728	Q4QKD1	−2.49	0.0007	

Genes that were either up or down regulated (≥1.5-fold plus P≤0.05, T-test and FDR≤0.05) when biofilms were formed in the presence of 170 ng/mL ampicillin are listed. Eight genes were up-regulated and 51 were down-regulated. Of the eight up-regulated genes, 5 were involved in carbohydrate metabolism and were enzymes involved in glycogen processing. The down-regulated genes were involved in a wide range of metabolic processes and 18 genes were unannotated (hypothetical).

### Glycogen was detected in ampicillin-treated NTHi biofilms

A commercial glycogen assay applied to four replicates of ampicillin-treated NTHi biofilms detected up to 0.68 µg of glycogen per mg of biofilm (0.019, 0.028, 0.41 and 0.68 µg/mg, with a mean of 0.14 µg per mg of biofilm (SD = 0.1). The lowest calibration point was 0.02 µg on the calibration graph. Glycogen was not detected at any level in four replicate untreated NTHi biofilms (data not shown).

**Figure 9:**
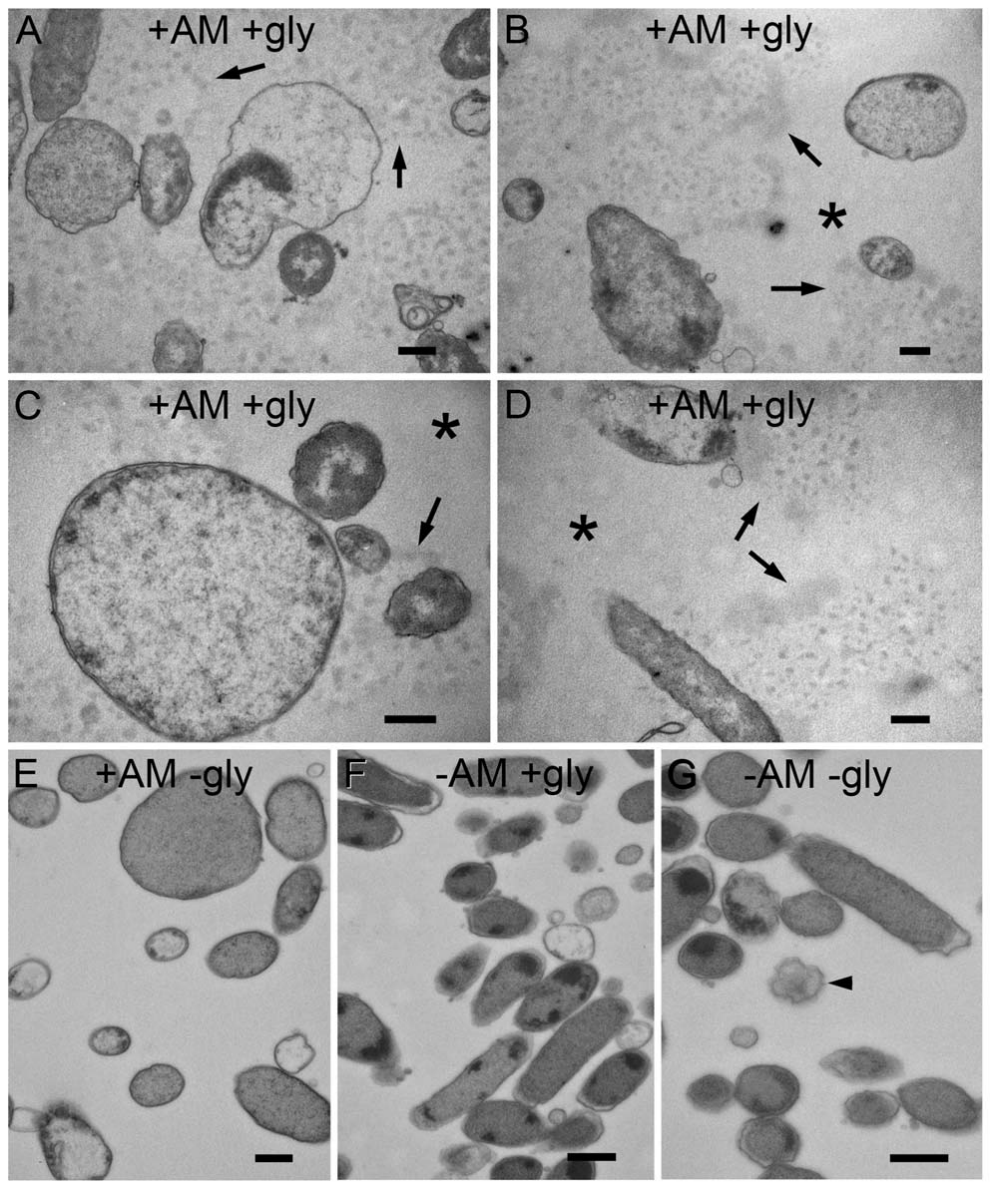
Ultrastructural visualization of glycogen in PittGG biofilms. Resin-embedded thin sections of PittGG biofilms formed in the presence or absence of ampicillin (+/− Am) and treated with a glycogen stain or a no stain control (+/− gly). A–D Biofilms with ampicillin, sections stained for glycogen (periodic acid and sodium chlorite). The glycogen stain reacted with an extracellular granular substance (arrows) that was associated with biofilm bacteria. Exposure to antibiotic resulted in a bacterial size increase with some bacteria (A–C). Extracellular areas that were not in close proximity to bacteria cells (asterisk) did not contain the extracellular granular substance (B–D). E. Biofilm with antibiotic but only treated with sodium chlorite did not reveal extracellular granular substance. Large bacterial cells were present. F. Biofilm with no antibiotic, sections stained for glycogen. No extracellular granular substance was detected and bacterial cells had a normal diameter (approx. 500 nm). Some lysed cells were detected. G. Biofilm with no antibiotic, treated with sodium chlorite (no periodic acid). Bacterial cells appear normal with a few dying cells present (arrowhead). Scale bars = 500 nm.

Application of a modified Schiff stain to sections through ampicillin-treated NTHi biofilms revealed the presence of an extracellular granular substance that we concluded as being extracellular glycogen (Fig. 9). The presumed glycogen stain was associated with the biofilm bacteria and did not appear in regions of the section that were biofilm-free (data not shown). The Schiff stain contrasted material in the extracellular matrix and did not stain intracellular sites (Fig. 9).

Although it is possible for processing and staining protocols to produce these contrasting patterns on thin sections we were unable to replicate them on any control experiments. Ampicillin-treated biofilm that was not exposed to the staining reagents did not show contrasted extracellular material (Fig. 9E). Biofilms not exposed to ampicillin and either treated with the stain reagents (Fig. 9F) on not treated (Fig. 9G) also did not show any contrasted extracellular material. The contrasted extracellular material was only present in sections through ampicillin-treated NTHi biofilms treated with the modified Schiff stain.

Ampicillin-treated NTHi biofilms contained biofilm bacteria with variable sizes (Fig. 9). Regular sized bacteria (∼500 nm thickness) were intermingled with NTHi bacteria that were 3 to 5 times larger (Fig. 9), and more spherical in appearance. Although some of the larger bacteria had a damaged appearance, others were morphologically intact (Fig. 9). The presence of the larger bacteria in ampicillin-treated biofilms was independent of whether the sections had been stained for glycogen, and were absent from untreated NTHi biofilms (Fig. 9).

## Discussion

The ability of NTHi bacteria to form biofilms *in vitro* [50], [52], [56], [57] was exploited here to investigate the stimulatory effect of sub-inhibitory concentrations of beta-lactam antibiotics on biofilm formation. We have found that some beta-lactam antibiotics were able to stimulate, or increase, biofilm formation by some strains of NTHi bacteria. The amount of antibiotic able to stimulate biofilm formation was dependent on the antibiotic used as well as the strain of NTHi that was exposed to the antibiotic. There was no uniform response across strains, and some strains did not react to the presence of the antibiotics. The NTHi strains that reacted to the antibiotic did so at different concentrations and antibiotic resistance/sensitivity was not a predictor of how a particular strain would react to sub-inhibitory concentrations of antibiotic. The biofilm stimulatory effect occurred below the minimum inhibitory concentration, was observed in a strain that was antibiotic resistant (PittEE), and was not observed in a strain susceptible to amoxicillin and cefuroxime (PittAA).

Biofilm stimulation was detected using the crystal violet biofilm assay, which measures the amount of biological material sticking to the surface of a container after bacteria have been cultured in it. It is not clear if this assay is an estimator of an increase in biofilm biomass or if it detects an increased ability of the biofilm material to attach to the sides of the plastic wells. For this reason, we examined the NTHi biofilms using other assays to confirm that beta-lactam antibiotic exposure was increasing biofilm biomass. In the presence of ampicillin, two different strains of NTHi showed an increase in dry weight and total protein content. However, antibiotic exposure lowered the ratio of protein to biofilm mass. The antibiotic also resulted in a drop in the number of viable bacteria in the biofilms, an observation supported by light microscopy, which showed more dead bacteria in the treated biofilms. Our direct observation of living biofilms also demonstrated the presence of antibiotic to cause an increase in the thickness of the formed biofilms.

Protein content in biofilms has previously been reported to derive from lysed cells or be actively secreted by biofilm-forming bacteria [58]. Our observation of decreased protein in the ampicillin-exposed biofilms might suggest a down-regulation of protein synthesis, a speculation that was supported by the comparison of mRNA from untreated and antibiotic-treated biofilms. The increase in biofilm biomass could also be partially explained by our mRNA analysis, which showed an up-regulation of genes involved with glycogen production in the presence of antibiotic. Glycogen, being a multi-unit sugar, would not be detected in a protein assay but its presence could account for the increased biomass we observed.

The increase in biomass may offer an explanation for why the ampicillin-stimulated PittGG biofilm was less firmly attached to the plastic surface after drying for SEM examination. The added volume and biofilm bulk could make an already weakly attached biofilm more easily dislodge during specimen preparation. Although it is possible that attachment mechanisms of the biofilm or biofilm bacteria have been modified by exposure to antibiotic, any such modifications were not evident in the mRNA data we collected.

The loss of bacterial cells after antibiotic exposure may indicate the expected massive cell death, and subsequent lysis, that theoretically may influence biofilm formation by chemical and physical aggregation lacking a biological component [59]. However, it must be considered that the bacteria in our experiment are adopting the biofilm phenotype that is by definition less able to grow under laboratory conditions. Transformation into the biofilm phenotype would mean that the NTHi bacteria become less culturable and less likely to be detected in viability assays, a common phenomenon observed for biofilm bacteria [60].

The composition of the partition walls we observed in the biofilms at the ultrastructural level is still a mystery. Increased production of glycogen might suggest that as the source of the partition wall material, a speculation that is currently being tested. The TEM examination of ampicillin-treated biofilms to detect glycogen did not reveal cross sections of partition walls, rather the glycogen in the ECM appeared globular and in close proximity to bacterial cells. The loss of bacterial cells in biofilms exposed to antibiotic might be due to massive cell death, which would liberate cytoplasmic proteins into the ECM and which could aggregate around nucleation sites such as provided by extracellular DNA [61] and form specific structures such as partition walls. The partition walls we observed were similar to ultrastructural features observed in biofilms from other bacteria [62]–[65]. Although it is still unclear what these partitions are composed of, proteins and DNA are common components of the extracellular matrix of biofilms formed by NTHi [50], [66], [67] and other bacteria [61], [63], [68]–[77]. Extracellular protein has been shown to remain functional in NTHi biofilms, maintaining the structural integrity of extracellular DNA, and perhaps forming DNA nets [67]. It is possible that the partitions observed in maturing biofilms as well as biofilms formed in the presence of biofilm-stimulating antibiotics result from extracellular protein, carbohydrate or glycogen aggregating on nets of extracellular DNA [61].

We selected beta-lactam antibiotics to study because these are the class of antibiotics most commonly used to treat patients with otitis media [22]. Although we used a common biofilm assay that bears little similarity to biofilms that might be formed in otitis media patients (in that middle ear epithelial cells were absent) we think it was appropriate to begin with a simple system where conditions could be controlled. A move to more complex and sophisticated biologically relevant models can be made once basic mechanisms have been elucidated.

Amoxicillin concentrations that have an *in vitro* biofilm stimulatory effect are in the range of 230–450 ng/mL. These *in vitro* amounts are much lower than recorded peak concentrations of amoxicillin in the middle ear fluids taken from otitis media patients, where the mean concentration was 9.5 µg/mL [78]. However, amoxicillin concentrations in the middle ear fluids range from undetectable to 20.6 µg/mL [78], a range broad enough to fit our experimental concentrations.

Recommended treatment protocols for otitis media consist of oral administration of amoxicillin, or amoxicillin combined with clavulanic acid, a beta-lactamase inhibitor [3], [22]. Recurrence, or lack of resolution of the infection can then be treated with a course of a different antibiotic, such as cefuroxime. These treatment protocols are very similar to those recommended for treating acute bacterial rhinosinusitis [3]. Using our *in vitro* system, we performed a preliminary experiment to mimic the treatment protocol where bacteria are first exposed to a sub-inhibitory dose of one antibody and then challenged with a higher dose of cefuroxime, a common antibiotic used to treat recalcitrant NTHi infections. Both the 2019 and PittGG strains showed that biofilm formation is important for protecting bacteria from a subsequent high dose of cefuroxime. Amoxicillin exposure appeared to increase the resistance of 2019 biofilm bacteria to a subsequent lethal dose of cefuroxime. Ampicillin exposure protected PittGG biofilm bacteria from the subsequent cefuroxime exposure. A sub-inhibitory dose of beta-lactam antibiotic influencing a subsequent antibiotic challenge is an effect that might occur under conditions in patients undergoing treatment for otitis media. It is thus worth further investigation. The phenomenon of increased resistance to a second course of antibiotics may help explain the increased occurrence of acute otitis media caused by NTHi [5] after an initial antibiotic course of treatment.

Outpatient beta-lactam antibiotics are mostly orally administered. Thus, the pharmacological bioavailability of the antibiotics may not be homogeneous throughout the body, with local concentrations being influenced by body weight, percent fat, gender, and drug accessibility. Bacterial biofilms are present throughout the human body [79] so it is highly likely that antibiotic treatments will impact commensal bacteria in uninfected regions of the body. If NTHi bacteria in the body react to beta-lactam antibiotics in a similar way to our *in vitro* observations, it is possible that an immediate and less desirable side effect of beta-lactam treatment of otitis media would be to stimulate bacterial attachment to surfaces within the body. Such a stimulus for attachment may signal a change in the NTHi causing it to become a pathogenic form. As such, these biofilm bacteria could then become a source of future chronic infections. Beta-lactam-treated patients would be expected to have an increased risk of recurrent infection. In fact, observations of otitis media patients do indicate an increased risk of recurrent otitis media after antibiotic treatment [23]. Increased occurrence of NTHi AOM is a consequence of previous antibiotic treatment [5].

While it has been reported that azithromycin can decrease established NTHi biofilms and inhibit NTHi biofilm formation [80], there appear to be few studies examining the effects of antibiotics stimulating NTHi biofilm formation. Antibiotics have been reported to stimulate biofilm formation in *Staphylococcus epidermidis* [28]–[34], *Pseudomonas aeruginosa* [35], [36], and *Escherichia coli* [39]. These reports, as well as the findings we report in this paper, all support the recently proposed theory that antibiotics may act as inter-microbial signaling molecules [35], [81] enabling microorganisms to communicate with each other.

## Conclusions

We have shown that sub-inhibitory concentrations of amoxicillin, ampicillin and cefuroxime (beta-lactam antibiotics) will stimulate NTHi bacteria to produce more biofilm biomass even as the numbers of culturable bacteria decline and as the biofilm protein content decreases. Biofilm bacteria were protected from the toxic effects of a high dose of cefuroxime. The biofilms formed in the presence of sub-inhibitory concentrations of antibiotic were thicker, had altered ultrastructure and contained increased numbers of dead bacteria. Sub-inhibitory concentrations of antibiotic up-regulated genes involved with glycogen biosynthesis, and glycogen was detected in antibiotic-treated biofilms. Genes encoding transporters and one gene involved with pyrimidine metabolism were also up-regulated. Surprisingly, genes involved with stress responses were not up-regulated. A total of 51 genes were down-regulated and were involved in a variety of metabolic processes, including cell-wall biosynthesis. Our results suggest that exposure to sub-inhibitory concentrations of beta-lactam antibiotic cause NTHi bacteria to increase glycogen synthesis while turning down a broad range of other metabolic processes. Beta-lactam-stimulated NTHi biofilms may protect embedded bacteria from subsequent high concentrations of cefuroxime.

## Supporting Information

Table S1 A) Statistical analysis for crystal violet assays in Fig. 2 and Fig. 3 comparing biofilm formation with sBHI-no bacteria control (numbers in parentheses indicate no significance). B) Statistical analysis for crystal violet assays in Fig. 2 and Fig. 3 comparing biofilm-stimulating effects of beta-lactam antibiotics.
(DOCX)

Table S2 **A**) NTHi sensitivity to Sensi-Disc Antimicrobial Susceptibility Test Discs. Reaction to antibiotic classified using manufacturers recommendation: R = Resistant, S = Susceptible, I – Intermediate. NTHi strains 49249 and 49766 are resistant and susceptible controls respectively. B) Minimum Inhibitory Concentrations (MIC) and Minimum Bactericidal Concentrations (MBC) in µg/mL antibiotic.
(DOCX)

Table S3 Statistical analysis for Figure 7: NTHi survival in 10 mg/mL cefuroxime after initial beta-lactam exposure. Key: AMP = ampicillin; Amox = amoxicillin; Cef = cefuroxime.
(DOCX)

Table S4 Significant gene list with all probe IDs identified by microarray analysis in the PittGG biofilm bacteria (NTHi) exposed to 170 ng/mL ampicillin.
(DOCX)
