# Correction: Beta- Lactam Antibiotics Stimulate Biofilm Formation in Non-Typeable *Haemophilus influenzae* by Up-Regulating Carbohydrate Metabolism

**DOI:** 10.1371/journal.pone.0115784

**Published:** 2014-12-11

**Authors:** The PLOS ONE Staff

There is an error in Table 1. The header on the second half of the table reads “Upregulated Genes” and it should read “Downregulated Genes.” The authors have provided the corrected table below.

**Table 1:** Significant genes identified by microarray transcriptomic analysis in the biofilm bacteria exposed to 170 ng/mL ampicillin.

UPREGULATED GENES						
	Gene Name	Ordered Locus	Uniprot	Fold Change	p-Value	Biological Process/function
**Carbohydrate Metabolism**						
ADP-glucose synthase	glgC	NTHI1807	Q4QK69	1.66	0.0008	glycogen biosynthesis
4-alpha-glucanotransferase	malQ	NTHI1810	Q4QK66	1.58	0.0003	glycosyltransferase
1,4-alpha-glucan branching enzyme	glgB	NTHI1809	Q4QK67	1.62	0.0007	glycogen biosynthesis
Glycogen operon protein GlgX	glgX	NTHI1808	Q4QK68	1.55	0.0009	glycogen catabolism
Glycogen synthase	glgA	NTHI1806	Q4QK70	1.86	0.0003	glycogen biosynthesis
**Transporter**						
Predicted cobalt transport protein		NTHI1421	Q4QL56	1.57	0.001	ABC transporter
**Pyrimidine Metabolism**						
Cytidine deaminase	cdd	NTHI1816	Q4QK60	1.65	0.0006	UMP synthesis
**Hypothetical Protein**						
Uncharacterized protein:pH regulation		NTHI0443	Q4QNL4	1.58	0.001	Na:H antiporter activity
**DOWNREGULATED GENES**						
**Amino-acid biosynthesis**						
Bifunctional protein FolD	folD	NTHI0864	Q4QMI7	-2.36	0.001	Amino-acid biosynthesis
Phosphoglycerol transferase-like protein		NTHI1918	Q4QJX3	-2.09	1E-05	Transferase
**Amino Acid Metabolism**						
Anthranilate synthase component I	trpE	NTHI1768	Q4QK98	-1.52	0.001	Lyase
Peptide methionine sulfoxide reductase	msaB	NTHI1677	Q4QKI0	-1.56	0.001	Oxidoreductase
Glutamate racemase	murI	NTHI205	Q4QJK7	-1.77	0.001	Cell wall biogenesis
**Folate Biosynthesis**						
dihydroneopterin aldolase	folB	NTHI0372	Q4QNS3	-1.71	0.001	Lyase
**Purine Metabolism**						
Bifunctional purine biosynthesis protein	purH	NTHI1051	Q4QM21	-1.98	0.001	Hydrolase
Phosphoribosylaminoimidazole carboxylase ATPase subunit	purK	NTHI1424	Q4QL53	-1.96	0.001	Lyase
N5-carboxyaminoimidazole ribonucleotide mutase	purE	NTHI1425	Q4QL52	-3.70	5E-05	Lyase
Phosphoribosylformylglycinamidine cyclo-ligase	purM	NTHI1704	Q4QKF3	-3.45	9E-05	Ligase
Phosphoribosylaminoimidazole-succinocarboxamide synthase	purC	NTHI2033	Q4QJM1	-2.50	0.0003	Ligase
Phosphoribosylglycinamide formyltransferase	purN	NTHI1706	Q4QKF2	-2.66	1E-04	Transferase
adenylosuccinase	purB	NTHI0758	Q4QMS8	-1.52	0.001	Lyase
**Regulation of transcription**						
BirA bifunctional protein	birA	NTHI0323	Q4QNW6	-2.63	0.0004	Ligase
Ribose operon repressor	rbsR	NTHI0634	Q4QN40	-1.62	0.001	DNA binding
Xylose operon regulatory protein	xylR	NTHI1273	Q4QLI5	-2.15	9E-05	DNA binding
**Toxin biosynthetic process**						
Colicin V production protein	cvpA	NTHI1377	Q4QL93	-1.92	0.001	Membrane protein
**Carbohydrate metabolism**						
D,D-heptose 1,7-bisphosphate phosphatase		NTHI0880	Q4QMH3	-3.21	0.0003	Hydrolase
Fructose-1,6-bisphosphatase	glpX	NTHI0789	Q4QMP9	-1.85	0.002	Hydrolase
Acetoacetate CoA transferase alpha subunit	atoD	NTHI0935	Q4QMC5	-1.75	0.0006	Transferase
**DNA replication**						
DNA polymerase III subunit epsilon	dnaQ	NTHI0223	Q4QP50	-2.29	7E-05	Transferase
Ribonuclease H	rnhA	NTHI0224	Q4QP49	-3.09	0.0001	Endonuclease
**Protein transporter**						
Competence protein E	comE	NTHI0560	Q4QNB0	-1.87	0.001	Protein secretion
Type IV pilin secretion protein	pilB	NTHI0408	Q4QNP5	-1.64	0.0006	
Type IV pilin secretion protein	pilC	NTHI0407	Q4QNP6	-1.74	0.001	
**Cellular cell wall organization**						
Membrane-bound lytic murein transglycosylase F	mltF	NTHI0338	Q4QNV4	-2.21	2E-04	
**Aminoacyl-tRNA biosynthesis**						
Lysine—tRNA ligase	genX	NTHI1003	Q4QM64	-1.60	0.001	
**ABC transporter**						
periplasmic oligopeptide-binding protein	oppA	NTHI1292	Q4QLH0	-1.74	0.0003	
ABC-type chelated iron transport system	hfeD	NTHI0477	Q4QNI3	-1.5	0.001	
**DNA packaging**						
Phage terminase large subunit		NTHI1741	Q4QKB9	-2.02	0.001	
**Protein deacetylation**						
NAD-dependent deacetylase sirtuin 5		NTHI1634	Q4QKL5	-2.80	2E-04	Hydrolase
**RNA repair**						
Multifunctional CCA protein	cca	NTHI1436	Q4QL41	-1.97	0.002	
**Amino acid transporter**						
Tryptophan-specific transport protein	mtr	NTHI0396	Q4QNQ4	-1.84	0.0002	
**Hypothetical Proteins**						
		NTHI0215	Q4QP56	-3.28	0.001	transmembrane transport
		NTHI0680	Q4QMZ6	-1.73	0.0004	
		NTHI0735	Q4QMU6	-1.64	0.0005	Predicated membrane protein
		NTHI1330	Q4QLD5	-1.64	0.001	
Conserved FAD/FMN-containing dehydrogenase		NTHI1331	Q4QLD4	-1.77	0.001	
		NTHI1505	Q4QKY1	-2.25	0.001	
		NTHI1511	Q4QKX6	-2.54	0.0007	DNA replication
		NTHI1528	Q4QKW1	-2.17	0.0005	
		NTHI1534	Q4QKV5	-1.84	0.002	
		NTHI1635	Q4QKL4	-4.08	0.001	Catalytic activity
		NTHI1721	Q4QKD8	-3.11	0.001	DNA binding
		NTHI1726	Q4QKD3	-1.92	0.0006	
		NTHI1735	Q4QKC5	-2.31	0.001	
		NTHI1736	Q4QKC4	-2.10	0.0008	Cell wall catabolism
		NTHI1738	Q4QKC2	-2.04	0.0003	
		NTHI1739	Q4QKC1	-2.12	0.0005	DNA binding
Putative recombination protein NinB	ninB	NTHI1727	Q4QKD2	-2.54	0.0006	
Putative recombination protein NinG	ninG	NTHI1728	Q4QKD1	-2.49	0.0007	

Genes that were either up or down regulated (≥1.5-fold plus P≤0.05, T-test and FDR ≤ 0.05) when biofilms were formed in the presence of 170 ng/mL ampicillin are listed. Eight genes were up-regulated and 51 were down-regulated. Of the eight up-regulated genes, 5 were involved in carbohydrate metabolism and were enzymes involved in glycogen processing. The down-regulated genes were involved in a wide range of metabolic processes and 18 genes were unannotated (hypothetical).
