# Supplementary material for: Beta- Lactam Antibiotics Stimulate Biofilm Formation in Non-Typeable Haemophilus influenzae by Up-Regulating Carbohydrate Metabolism
Source: PLoS One. 2014 Jul 9;9(7):e99204. doi: 10.1371/journal.pone.0099204 (PMC4090067; doi:10.1371/journal.pone.0099204)
Supplement: Table S1 — A) Statistical analysis for crystal violet assays in Fig. 2 and Fig. 3 comparing biofilm formation with sBHI-no bacteria control (numbers in parentheses indicate no significance). B) Statistical analysis for crystal violet assays in Fig. 2 and Fig. 3 comparing biofilm-stimulating effects of beta-lactam antibiotics. (DOCX) [file pone.0099204.s001.docx]

**Table S1**

**A p-values for Figure 2**

| NTHi  Strain | **p-values: Sterile BHI v. BHI plus NTHi** | | |
| --- | --- | --- | --- |
|  | Amoxicillin | Ampicillin | Cefuroxime |
| 2019 | 0.0354 | <0.0001 | <0.0001 |
| 9274 | (0.0882) | <0.0001 | 0.0002 |
| PittAA | (0.5170) | <0.0001 | <0.0001 |
| PittEE | 0.0027 | <0.0001 | <0.0001 |
| PittGG | (0.8798) | 0.0016 | <0.0001 |
| PittII | (0.0875) | <0.0001 | <0.0001 |

**p-value for Figure 3**

| Pitt GG |  |  | <0.0001 |
| --- | --- | --- | --- |

**B p-values for Figure 2**

| NTHi  Strain | **p-values: no antibiotic v. max stimulation** | | |
| --- | --- | --- | --- |
|  | Amoxicillin | Ampicillin | Cefuroxime |
| 2019 | (0.0921) | <0.0001 | <0.0001 |
| 9274 | 0.0002 | 0.0095 | <0.0001 |
| PittAA | 0.0003 | (0.241) | <0.0001 |
| PittEE | <0.0001 | <0.0001 | <0.0001 |
| PittGG | 0.0047 | 0.0336 | <0.0001 |
| PittII | <0.0001 | (0.0946) | <0.0001 |

**p-value for Figure 3**

| Pitt GG |  |  | <0.0001 |
| --- | --- | --- | --- |
