# Supplementary material for: Beta- Lactam Antibiotics Stimulate Biofilm Formation in Non-Typeable Haemophilus influenzae by Up-Regulating Carbohydrate Metabolism
Source: PLoS One. 2014 Jul 9;9(7):e99204. doi: 10.1371/journal.pone.0099204 (PMC4090067; doi:10.1371/journal.pone.0099204)
Supplement: Table S2 — A) NTHi sensitivity to Sensi-Disc Antimicrobial Susceptibility Test Discs. Reaction to antibiotic classified using manufacturers recommendation: R = Resistant, S = Susceptible, I – Intermediate. NTHi strains 49249 and 49766 are resistant and susceptible controls respectively. B) Minimum Inhibitory Concentrations (MIC) and Minimum Bactericidal Concentrations (MBC) in µg/mL antibiotic. (DOCX) [file pone.0099204.s002.docx]

**Table S2**

**A)**

|  | Antibiotic | | | |
| --- | --- | --- | --- | --- |
| NTHi Strain | Augmentin | Ampicillin | Cefuroxime | Vancomysin |
|  | Up to 10 µg | Up to 10 µg | Up to 30 µg | Up to 30 µg |
| 49249 | R | R | R | R |
| 49766 | S | S | S | R |
| 2019 | S | S | S | R |
| 9274 | S | S | S | R |
| PittAA | S | R | S | R |
| PittEE | R | R | I | R |
| PittII | S | S | S | R |
| PittGG | S | R | S | R |

**B)**

|  | Antibiotic | | | | | |
| --- | --- | --- | --- | --- | --- | --- |
|  | Amoxicillin | | Ampicillin | | Cefuroxime | |
| NTHi Strain | MIC | MBC | MIC | MBC | MIC | MBC |
| 2019 | 1.3 | 1.9 | 1.3 | 1.9 | 1.3 | 1.9 |
| 9274 | 75 | 200 | 75 | 200 | 1.3 | 1.9 |
| PittAA | 150 | >300 | 800 | 1,000 | 15.6 | 62.5 |
| PittEE | 50 | 50 | 6.25 | 6.25 | 1.3 | 1.9 |
| PittII | 150 | 300 | 150 | 300 | 62.5 | 62.5 |
| PittGG | 3.13 | 3.13 | 1.3 | 1.9 | 31.3 | 62.5 |
