# Supplementary material for: Beta- Lactam Antibiotics Stimulate Biofilm Formation in Non-Typeable Haemophilus influenzae by Up-Regulating Carbohydrate Metabolism
Source: PLoS One. 2014 Jul 9;9(7):e99204. doi: 10.1371/journal.pone.0099204 (PMC4090067; doi:10.1371/journal.pone.0099204)
Supplement: Table S3 — Statistical analysis for Figure 7: NTHi survival in 10 mg/mL cefuroxime after initial beta-lactam exposure. Key: AMP = ampicillin; Amox = amoxicillin; Cef = cefuroxime. (DOCX) [file pone.0099204.s003.docx]

**Table S3**

| **Day 1**  **Antibiotic Treatment** | **Day 2 Cefuroxime Treatment** | **Cell Count**  **2019** | **Cell Count**  **PittGG** | **Survival (%)**  **2019** | ***p*-value** | **Survival (%)**  **PittGG** | ***p*-value** |
| --- | --- | --- | --- | --- | --- | --- | --- |
| No treatment | NO | 820000 | 170000 | 56.1% |  | 23.5% |  |
|  | YES | 460000 | 40000 |  |  |  |  |
| 150 µg/ml ampicillin | NO | 570000 | 200000 | 28.1% | <0.0001 | 6.5% | <0.0001 |
|  | YES | 160000 | 13000 |  |  |  |  |
| 230 µg/ml amoxicillin | NO | 140000 | 31000 | 85.7% | 0.0059 | 19.7% | 0.0072 |
|  | YES | 120000 | 6100 |  |  |  |  |
| 170 µg/ml cefuroxime | NO | 620000 | 150000 | 60.0% | 0.2611 | 23.3% | 0.9023 |
|  | YES | 370000 | 35000 |  |  |  |  |
