# Supplementary material for: Beta- Lactam Antibiotics Stimulate Biofilm Formation in Non-Typeable Haemophilus influenzae by Up-Regulating Carbohydrate Metabolism
Source: PLoS One. 2014 Jul 9;9(7):e99204. doi: 10.1371/journal.pone.0099204 (PMC4090067; doi:10.1371/journal.pone.0099204)
Supplement: Table S4 — Significant gene list with all probe IDs identified by microarray analysis in the PittGG biofilm bacteria (NTHi) exposed to 170 ng/mL ampicillin. (DOCX) [file pone.0099204.s004.docx]

**Table S4**

| **Pathway** | **Probeset ID** | **Locus** | **Uniprot** | **Gene** | **Function** | **Fold-Change** | **P-value** | **Average P-value** | **Biological process** |
| --- | --- | --- | --- | --- | --- | --- | --- | --- | --- |
|  |  |  |  |  |  |  |  |  |  |
| **Upregulated** |  |  |  |  |  |  |  |  |  |
|  |  |  |  |  |  |  |  |  |  |
| **Carbohydrate**  **metabolism** | NTHI1807111200001576.6 | NTHI1807 | Q4QK69 | glgC | ADP-glucose synthase | 1.71 | 0.0011 |  | glycogen biosynthesis |
|  | NTHI1807111200001576.2 |  |  |  |  | 1.70 | 0.0004 |  |  |
|  | NTHI1807111200001576.5 |  |  |  |  | 1.69 | 0.0010 |  |  |
|  | NTHI1807111200001576.4 |  |  |  |  | 1.67 | 0.0010 |  |  |
|  | NTHI1807111200001576.1 |  |  |  |  | 1.66 | 0.0001 |  |  |
|  | NTHI1807111200001576.3 |  |  |  |  | 1.64 | 0.0005 |  |  |
|  | NTHI1807111200001576.7 |  |  |  |  | 1.60 | 0.0017 | 0.0008 |  |
|  |  |  |  |  |  | *Mean 1.66* |  |  |  |
|  | NTHI1810111200001579.7 | NTHI1810 | Q4QK66 | malQ | amylomaltase | 1.62 | 0.0010 |  | glycosyltransferase |
|  | NTHI1810111200001579.3 |  |  |  |  | 1.62 | 0.0002 |  |  |
|  | NTHI1810111200001579.1 |  |  |  |  | 1.61 | 0.0001 |  |  |
|  | NTHI1810111200001579.6 |  |  |  |  | 1.58 | 0.0004 |  |  |
|  | NTHI1810111200001579.2 |  |  |  |  | 1.57 | 0.0001 |  |  |
|  | NTHI1810111200001579.4 |  |  |  |  | 1.53 | 0.0002 |  |  |
|  | NTHI1810111200001579.5 |  |  |  |  | 1.51 | 0.0004 | 0.0003 |  |
|  |  |  |  |  |  | *Mean 1.58* |  |  |  |
|  | NTHI1809111200001578.1 | NTHI1809 | Q4QK67 | glgB | glycogen branching enzyme | 1.64 | 0.0005 |  | glycogen biosynthesis |
|  | NTHI1809111200001578.2 |  |  |  |  | 1.63 | 0.0006 |  |  |
|  | NTHI1809111200001578.6 |  |  |  |  | 1.61 | 0.0008 |  |  |
|  | NTHI1809111200001578.3 |  |  |  |  | 1.59 | 0.0008 | 0.0007 |  |
|  |  |  |  |  |  | *Mean 1.62* |  |  |  |
|  | NTHI1808111200001577.3 | NTHI1808 | Q4QK68 | glgX | glycogen operon protein GlgX | 1.58 | 0.0009 |  | glycogen catabolism |
|  | NTHI1808111200001577.2 |  |  |  |  | 1.57 | 0.0005 |  |  |
|  | NTHI1808111200001577.5 |  |  |  |  | 1.54 | 0.0010 |  |  |
|  | NTHI1808111200001577.6 |  |  |  |  | 1.54 | 0.0010 |  |  |
|  | NTHI1808111200001577.1 |  |  |  |  | 1.53 | 0.0005 |  |  |
|  | NTHI1808111200001577.7 |  |  |  |  | 1.52 | 0.0015 | 0.0009 |  |
|  |  |  |  |  |  | *Mean 1.55* |  |  |  |
|  | NTHI1806111200001575.5 | NTHI1806 | Q4QK70 | glgA | Starch [bacterial glycogen] synthase | 1.96 | 0.0002 |  | glycogen biosynthesis |
|  | NTHI1806111200001575.6 |  |  |  |  | 1.93 | 0.0005 |  |  |
|  | NTHI1806111200001575.2 |  |  |  |  | 1.85 | 0.0000 |  |  |
|  | NTHI1806111200001575.4 |  |  |  |  | 1.84 | 0.0002 |  |  |
|  | NTHI1806111200001575.1 |  |  |  |  | 1.83 | 0.0000 |  |  |
|  | NTHI1806111200001575.3 |  |  |  |  | 1.81 | 0.0001 |  |  |
|  | NTHI1806111200001575.7 |  |  |  |  | 1.80 | 0.0010 | 0.0003 |  |
|  |  |  |  |  |  | *Mean 1.86* |  |  |  |
|  |  |  |  |  |  |  |  |  |  |
| **ABC transporter** | NTHI1421111200001239.6 | NTHI1421 | Q4QL56 |  | cobalt ABC transporter permease | 1.57 | 0.0013 |  |  |
|  | NTHI1421111200001239.2 |  |  |  |  | 1.56 | 0.0011 | 0.001 |  |
|  |  |  |  |  |  | *Mean 1.57* |  |  |  |
|  |  |  |  |  |  |  |  |  |  |
| **Pyrimidine**  **metabolism** | NTHI1816111200001585.3 | NTHI1816 | Q4QK60 | cdd | cytidine aminohydrolase | 1.65 | 0.0006 | 0.0006 | UMP synthesis |
|  |  |  |  |  |  |  |  |  |  |
|  |  |  |  |  |  |  |  |  |  |
| **Unknown** | NTHI0443111200000381.3 | NTHI0443 | Q4QNL4 |  | hypothetical protein | 1.58 | 0.0015 | 0.001 | pH regulation |
|  |  |  |  |  |  |  |  |  |  |
|  |  |  |  |  |  |  |  |  |  |
| **Downregulated** |  |  |  |  |  |  |  |  |  |
|  |  |  |  |  |  |  |  |  |  |
| **Amino-acid**  **biosynthesis** | NTHI0864111200000758.4 | NTHI0864 | Q4QMI7 | folD | 5,10-methylene-tetrahydrofolate cyclohydrolase | -2.30 | 0.0015 |  |  |
|  | NTHI0864111200000758.5 |  |  |  |  | -2.30 | 0.0015 |  |  |
|  | NTHI0864111200000758.6 |  |  |  |  | -2.33 | 0.0016 |  |  |
|  | NTHI0864111200000758.1 |  |  |  |  | -2.35 | 0.0012 |  |  |
|  | NTHI0864111200000758.3 |  |  |  |  | -2.49 | 0.0014 | 0.001 |  |
|  |  |  |  |  |  | *Mean -2.36* |  |  |  |
|  |  |  |  |  |  |  |  |  |  |
| **Transferase** | NTHI1918111200001672.2 | NTHI1918 | Q4QJX3 |  | phosphoglycerol transferase-like protein | -2.03 | 0.00000 |  |  |
|  | NTHI1918111200001672.3 |  |  |  |  | -2.06 | 0.00000 |  |  |
|  | NTHI1918111200001672.6 |  |  |  |  | -2.07 | 0.00001 |  |  |
|  | NTHI1918111200001672.5 |  |  |  |  | -2.07 | 0.00001 |  |  |
|  | NTHI1918111200001672.1 |  |  |  |  | -2.08 | 0.00000 |  |  |
|  | NTHI1918111200001672.7 |  |  |  |  | -2.14 | 0.00007 |  |  |
|  | NTHI1918111200001672.4 |  |  |  |  | -2.19 | 0.00001 | 0.00001 |  |
|  |  |  |  |  |  | *Mean -2.09* |  |  |  |
|  |  |  |  |  |  |  |  |  |  |
| **Amino Acid**  **Metabolism** | NTHI1768111200001547.4 | NTHI1768 | Q4QK98 | trpE | anthranilate synthase component I | -1.51 | 0.0010 |  |  |
|  | NTHI1768111200001547.3 |  |  |  |  | -1.51 | 0.0007 |  |  |
|  | NTHI1768111200001547.7 |  |  |  |  | -1.52 | 0.0013 |  |  |
|  | NTHI1768111200001547.6 |  |  |  |  | -1.53 | 0.0010 |  |  |
|  | NTHI1768111200001547.1 |  |  |  |  | -1.53 | 0.0006 | 0.001 |  |
|  |  |  |  |  |  | *Mean -1.52* |  |  |  |
|  | NTHI1677111200001465.6 | NTHI1677 | Q4QKI0 | msaB | peptide methionine sulfoxide reductase | -1.51 | 0.0013 |  |  |
|  | NTHI1677111200001465.3 |  |  |  |  | -1.55 | 0.0011 |  |  |
|  | NTHI1677111200001465.5 |  |  |  |  | -1.56 | 0.0012 |  |  |
|  | NTHI1677111200001465.7 |  |  |  |  | -1.57 | 0.0016 |  |  |
|  | NTHI1677111200001465.1 |  |  |  |  | -1.60 | 0.0008 | 0.001 |  |
|  |  |  |  |  |  | *Mean -1.56* |  |  |  |
|  | NTHI2050111200001788.3 | NTHI2050 | Q4QJK7 | murI | glutamate racemase | -1.72 | 0.0016 |  |  |
|  | NTHI2050111200001788.1 |  |  |  |  | -1.81 | 0.0013 | 0.001 |  |
|  |  |  |  |  |  | *Mean -1.77* |  |  |  |
|  |  |  |  |  |  |  |  |  |  |
| **Folate**  **biosynthesis** | NTHI0372111200000322.7 | NTHI0372 | Q4QNS3 | folB | dihydroneopterin aldolase | -1.68 | 0.0015 |  |  |
|  | NTHI0372111200000322.1 |  |  |  |  | -1.71 | 0.0002 |  |  |
|  | NTHI0372111200000322.4 |  |  |  |  | -1.71 | 0.0009 |  |  |
|  | NTHI0372111200000322.6 |  |  |  |  | -1.71 | 0.0012 |  |  |
|  | NTHI0372111200000322.5 |  |  |  |  | -1.72 | 0.0009 |  |  |
|  | NTHI0372111200000322.2 |  |  |  |  | -1.73 | 0.0008 | 0.001 |  |
|  |  |  |  |  |  | *Mean -1.71* |  |  |  |
|  |  |  |  |  |  |  |  |  |  |
| **Purine**  **metabolism** | NTHI1051111200000924.4 | NTHI1051 | Q4QM21 | purH | AICAR transformylase | -1.79 | 0.0006 |  | Purine biosynthesis |
|  | NTHI1051111200000924.5 |  |  |  |  | -1.88 | 0.0010 |  |  |
|  | NTHI1051111200000924.1 |  |  |  |  | -1.90 | 0.0003 |  |  |
|  | NTHI1051111200000924.2 |  |  |  |  | -1.97 | 0.0004 |  |  |
|  | NTHI1051111200000924.7 |  |  |  |  | -2.03 | 0.0011 |  |  |
|  | NTHI1051111200000924.6 |  |  |  |  | -2.13 | 0.0010 |  |  |
|  | NTHI1051111200000924.3 |  |  |  |  | -2.14 | 0.0005 | 0.001 |  |
|  |  |  |  |  |  | *Mean -1.98* |  |  |  |
|  |  |  |  |  |  |  |  |  |  |
|  | NTHI1424111200001242.1 | NTHI1424 | Q4QL53 | purK | AIR carboxylase | -1.96 | 0.0009 | 0.001 |  |
|  |  |  |  |  |  |  |  |  |  |
|  | NTHI1425111200001243.5 | NTHI1425 | Q4QL52 | purE | AIR carboxylase | -3.51 | 0.0001 |  | Purine biosynthesis |
|  | NTHI1425111200001243.7 |  |  |  |  | -3.58 | 0.0001 |  |  |
|  | NTHI1425111200001243.2 |  |  |  |  | -3.69 | 0.0000 |  |  |
|  | NTHI1425111200001243.6 |  |  |  |  | -3.72 | 0.0001 |  |  |
|  | NTHI1425111200001243.3 |  |  |  |  | -3.77 | 0.0000 |  |  |
|  | NTHI1425111200001243.1 |  |  |  |  | -3.82 | 0.0000 |  |  |
|  | NTHI1425111200001243.4 |  |  |  |  | -3.83 | 0.0000 | 0.00005 |  |
|  |  |  |  |  |  | *Mean -3.70* |  |  |  |
|  | NTHI1704111200001492.7 | NTHI1704 | Q4QKF3 | purM | AIR synthase | -3.35 | 0.0001 |  | Purine biosynthesis |
|  | NTHI1704111200001492.6 |  |  |  |  | -3.40 | 0.0001 |  |  |
|  | NTHI1704111200001492.2 |  |  |  |  | -3.41 | 0.0000 |  |  |
|  | NTHI1704111200001492.5 |  |  |  |  | -3.45 | 0.0001 |  |  |
|  | NTHI1704111200001492.4 |  |  |  |  | -3.45 | 0.0001 |  |  |
|  | NTHI1704111200001492.3 |  |  |  |  | -3.52 | 0.0001 |  |  |
|  | NTHI1704111200001492.1 |  |  |  |  | -3.55 | 0.0000 | 0.00009 |  |
|  |  |  |  |  |  | *Mean -3.45* |  |  |  |
|  | NTHI2033111200001774.5 | NTHI2033 | Q4QJM1 | purC | SAICAR synthetase | -2.44 | 0.0003 |  | Purine biosynthesis |
|  | NTHI2033111200001774.6 |  |  |  |  | -2.44 | 0.0003 |  |  |
|  | NTHI2033111200001774.4 |  |  |  |  | -2.50 | 0.0003 |  |  |
|  | NTHI2033111200001774.3 |  |  |  |  | -2.50 | 0.0003 |  |  |
|  | NTHI2033111200001774.2 |  |  |  |  | -2.53 | 0.0002 |  |  |
|  | NTHI2033111200001774.7 |  |  |  |  | -2.54 | 0.0005 |  |  |
|  | NTHI2033111200001774.1 |  |  |  |  | -2.55 | 0.0002 | 0.0003 |  |
|  |  |  |  |  |  | *Mean -2.50* |  |  |  |
|  | NTHI1706111200001493.7 | NTHI1706 | Q4QKF2 | purN | GAR transformylase | -2.49 | 0.0002 |  | IMP biosynthetic process |
|  | NTHI1706111200001493.4 |  |  |  |  | -2.63 | 0.0001 |  |  |
|  | NTHI1706111200001493.5 |  |  |  |  | -2.66 | 0.0001 |  |  |
|  | NTHI1706111200001493.2 |  |  |  |  | -2.66 | 0.0000 |  |  |
|  | NTHI1706111200001493.6 |  |  |  |  | -2.66 | 0.0001 |  |  |
|  | NTHI1706111200001493.1 |  |  |  |  | -2.72 | 0.0000 |  |  |
|  | NTHI1706111200001493.3 |  |  |  |  | -2.81 | 0.0001 | 0.0001 |  |
|  |  |  |  |  |  | *Mean -2.66* |  |  |  |
|  | NTHI0758111200000667.6 | NTHI0758 | Q4QMS8 | purB | adenylosuccinase | -1.50 | 0.0011 |  | IMP biosynthetic process |
|  | NTHI0758111200000667.7 |  |  |  |  | -1.52 | 0.0015 |  |  |
|  | NTHI0758111200000667.4 |  |  |  |  | -1.52 | 0.0007 |  |  |
|  | NTHI0758111200000667.5 |  |  |  |  | -1.52 | 0.0008 |  |  |
|  | NTHI0758111200000667.2 |  |  |  |  | -1.52 | 0.0003 | 0.001 |  |
|  |  |  |  |  |  | *Mean -1.52* |  |  |  |
|  |  |  |  |  |  |  |  |  |  |
| **Regulation of**  **transcription** | NTHI0323111200000279.6 | NTHI0323 | Q4QNW6 | birA | Biotin--protein ligase | -2.55 | 0.0005 |  |  |
|  | NTHI0323111200000279.1 |  |  |  |  | -2.61 | 0.0003 |  |  |
|  | NTHI0323111200000279.4 |  |  |  |  | -2.61 | 0.0003 |  |  |
|  | NTHI0323111200000279.7 |  |  |  |  | -2.61 | 0.0006 |  |  |
|  | NTHI0323111200000279.2 |  |  |  |  | -2.65 | 0.0003 |  |  |
|  | NTHI0323111200000279.3 |  |  |  |  | -2.65 | 0.0003 |  |  |
|  | NTHI0323111200000279.5 |  |  |  |  | -2.72 | 0.0005 | 0.0004 |  |
|  |  |  |  |  |  | *Mean -2.63* |  |  |  |
|  | NTHI0634111200000555.1 | NTHI0634 | Q4QN40 | rbsR | ribose operon repressor | -1.56 | 0.0003 |  |  |
|  | NTHI0634111200000555.2 |  |  |  |  | -1.61 | 0.0006 |  |  |
|  | NTHI0634111200000555.7 |  |  |  |  | -1.61 | 0.0014 |  |  |
|  | NTHI0634111200000555.4 |  |  |  |  | -1.63 | 0.0010 |  |  |
|  | NTHI0634111200000555.3 |  |  |  |  | -1.63 | 0.0007 |  |  |
|  | NTHI0634111200000555.6 |  |  |  |  | -1.64 | 0.0013 |  |  |
|  | NTHI0634111200000555.5 |  |  |  |  | -1.66 | 0.0013 | 0.001 |  |
|  |  |  |  |  |  | *Mean -1.62* |  |  |  |
|  | NTHI1273111200001110.3 | NTHI1273 | Q4QLI5 | xylR | xylose operon regulatory protein | -2.12 | 0.0000 |  |  |
|  | NTHI1273111200001110.7 |  |  |  |  | -2.13 | 0.0002 |  |  |
|  | NTHI1273111200001110.2 |  |  |  |  | -2.14 | 0.0000 |  |  |
|  | NTHI1273111200001110.1 |  |  |  |  | -2.15 | 0.0000 |  |  |
|  | NTHI1273111200001110.4 |  |  |  |  | -2.15 | 0.0001 |  |  |
|  | NTHI1273111200001110.6 |  |  |  |  | -2.15 | 0.0001 |  |  |
|  | NTHI1273111200001110.5 |  |  |  |  | -2.22 | 0.0001 | 0.00009 |  |
|  |  |  |  |  |  | *Mean -2.15* |  |  |  |
|  |  |  |  |  |  |  |  |  |  |
| **toxin biosynthetic**  **process** | NTHI1377111200001202.5 | NTHI1377 | Q4QL93 | cvpA | colicin V production protein | -1.88 | 0.0011 |  |  |
|  | NTHI1377111200001202.3 |  |  |  |  | -1.88 | 0.0011 |  |  |
|  | NTHI1377111200001202.7 |  |  |  |  | -1.89 | 0.0015 |  |  |
|  | NTHI1377111200001202.4 |  |  |  |  | -1.92 | 0.0011 |  |  |
|  | NTHI1377111200001202.6 |  |  |  |  | -1.94 | 0.0014 |  |  |
|  | NTHI1377111200001202.1 |  |  |  |  | -1.95 | 0.0007 |  |  |
|  | NTHI1377111200001202.2 |  |  |  |  | -1.96 | 0.0009 | 0.001 |  |
|  |  |  |  |  |  | *Mean -1.92* |  |  |  |
|  |  |  |  |  |  |  |  |  |  |
| **Carbohydrate**  **metabolism** | NTHI0880111200000772.4 | NTHI0880 | Q4QMH3 | gmhB family | D,D-heptose 1,7-bisphosphate phosphatase | -3.09 | 0.0004 |  |  |
|  | NTHI0880111200000772.1 |  |  |  |  | -3.20 | 0.0002 |  |  |
|  | NTHI0880111200000772.2 |  |  |  |  | -3.20 | 0.0003 |  |  |
|  | NTHI0880111200000772.6 |  |  |  |  | -3.20 | 0.0004 |  |  |
|  | NTHI0880111200000772.5 |  |  |  |  | -3.25 | 0.0004 |  |  |
|  | NTHI0880111200000772.7 |  |  |  |  | -3.25 | 0.0004 |  |  |
|  | NTHI0880111200000772.3 |  |  |  |  | -3.30 | 0.0003 | 0.0003 |  |
|  |  |  |  |  |  | *Mean -3.21* |  |  |  |
|  | NTHI0789111200000696.3 | NTHI0789 | Q4QMP9 | glpX | FBPase II glpX | -1.83 | 0.0015 |  |  |
|  | NTHI0789111200000696.4 |  |  |  |  | -1.83 | 0.0015 |  |  |
|  | NTHI0789111200000696.7 |  |  |  |  | -1.88 | 0.0015 | 0.002 |  |
|  |  |  |  |  |  | *Mean -1.85* |  |  |  |
|  | NTHI0935111200000820.4 | NTHI0935 | Q4QMC5 | atoD | acetoacetate CoA transferase alpha subunit | -1.72 | 0.0004 |  |  |
|  | NTHI0935111200000820.6 |  |  |  |  | -1.73 | 0.0008 |  |  |
|  | NTHI0935111200000820.2 |  |  |  |  | -1.75 | 0.0004 |  |  |
|  | NTHI0935111200000820.7 |  |  |  |  | -1.76 | 0.0008 |  |  |
|  | NTHI0935111200000820.5 |  |  |  |  | -1.77 | 0.0006 |  |  |
|  | NTHI0935111200000820.3 |  |  |  |  | -1.77 | 0.0004 | 0.0006 |  |
|  |  |  |  |  |  | *Mean -1.75* |  |  |  |
|  |  |  |  |  |  |  |  |  |  |
| **DNA replication** | NTHI0223111200000195.4 | NTHI0223 | Q4QP50 | dnaQ | DNA polymerase III subunit epsilon | -2.20 | 0.0001 |  |  |
|  | NTHI0223111200000195.2 |  |  |  |  | -2.25 | 0.0000 |  |  |
|  | NTHI0223111200000195.1 |  |  |  |  | -2.25 | 0.0000 |  |  |
|  | NTHI0223111200000195.6 |  |  |  |  | -2.30 | 0.0001 |  |  |
|  | NTHI0223111200000195.7 |  |  |  |  | -2.31 | 0.0001 |  |  |
|  | NTHI0223111200000195.5 |  |  |  |  | -2.35 | 0.0001 |  |  |
|  | NTHI0223111200000195.3 |  |  |  |  | -2.38 | 0.0000 | 0.00007 |  |
|  |  |  |  |  |  | *Mean -2.29* |  |  |  |
|  | NTHI0224111200000196.6 | NTHI0224 | Q4QP49 | rnhA | RNase HI | -3.04 | 0.0001 |  |  |
|  | NTHI0224111200000196.5 |  |  |  |  | -3.05 | 0.0001 |  |  |
|  | NTHI0224111200000196.1 |  |  |  |  | -3.08 | 0.0001 |  |  |
|  | NTHI0224111200000196.7 |  |  |  |  | -3.09 | 0.0002 |  |  |
|  | NTHI0224111200000196.2 |  |  |  |  | -3.11 | 0.0001 |  |  |
|  | NTHI0224111200000196.3 |  |  |  |  | -3.11 | 0.0001 |  |  |
|  | NTHI0224111200000196.4 |  |  |  |  | -3.12 | 0.0001 | 0.0001 |  |
|  |  |  |  |  |  | *Mean -3.09* |  |  |  |
|  |  |  |  |  |  |  |  |  |  |
| **Protein**  **transporter** | NTHI0560111200000485.7 | NTHI0560 | Q4QNB0 | comE | DNA transformation protein comE | -1.83 | 0.0015 |  |  |
|  | NTHI0560111200000485.1 |  |  |  |  | -1.87 | 0.0006 |  |  |
|  | NTHI0560111200000485.5 |  |  |  |  | -1.88 | 0.0011 |  |  |
|  | NTHI0560111200000485.3 |  |  |  |  | -1.90 | 0.0011 | 0.001 |  |
|  |  |  |  |  |  | *Mean -1.87* |  |  |  |
|  | NTHI0408111200000350.1 | NTHI0408 | Q4QNP5 | pilB | type IV pilin secretion protein | -1.55 | 0.0004 |  |  |
|  | NTHI0408111200000350.5 |  |  |  |  | -1.61 | 0.0009 |  |  |
|  | NTHI0408111200000350.3 |  |  |  |  | -1.68 | 0.0007 |  |  |
|  | NTHI0408111200000350.2 |  |  |  |  | -1.73 | 0.0004 | 0.0006 |  |
|  |  |  |  |  |  | *Mean -1.64* |  |  |  |
|  | NTHI0407111200000349.1 | NTHI0407 | Q4QNP6 | pilC | type IV pilin secretion protein | -1.74 | 0.0013 | 0.001 |  |
|  |  |  |  |  |  |  |  |  |  |
|  |  |  |  |  |  |  |  |  |  |
| **Cellular cell wall organization** | NTHI0338111200000291.3 | NTHI0338 | Q4QNV4 | mltF | transglycosylase | -2.14 | 0.0001 |  |  |
|  | NTHI0338111200000291.7 |  |  |  |  | -2.15 | 0.0003 |  |  |
|  | NTHI0338111200000291.2 |  |  |  |  | -2.21 | 0.0001 |  |  |
|  | NTHI0338111200000291.5 |  |  |  |  | -2.23 | 0.0002 |  |  |
|  | NTHI0338111200000291.6 |  |  |  |  | -2.23 | 0.0003 |  |  |
|  | NTHI0338111200000291.4 |  |  |  |  | -2.27 | 0.0002 |  |  |
|  | NTHI0338111200000291.1 |  |  |  |  | -2.28 | 0.0000 | 0.0002 |  |
|  |  |  |  |  |  | *Mean -2.21* |  |  |  |
|  |  |  |  |  |  |  |  |  |  |
| **Aminoacyl-tRNA biosynthesis** | NTHI1003111200000881.6 | NTHI1003 | Q4QM64 | genX | Lysine--tRNA ligase | -1.56 | 0.0017 |  |  |
|  | NTHI1003111200000881.4 |  |  |  |  | -1.57 | 0.0017 |  |  |
|  | NTHI1003111200000881.3 |  |  |  |  | -1.63 | 0.0007 |  |  |
|  | NTHI1003111200000881.1 |  |  |  |  | -1.64 | 0.0003 | 0.001 |  |
|  |  |  |  |  |  | *Mean -1.60* |  |  |  |
|  |  |  |  |  |  |  |  |  |  |
| **ABC Transporter** | NTHI1292111200001125.1 | NTHI1292 | Q4QLH0 | oppA | periplasmic oligopeptide-binding protein | -1.68 | 0.0001 |  |  |
|  | NTHI1292111200001125.3 |  |  |  |  | -1.71 | 0.0001 |  |  |
|  | NTHI1292111200001125.6 |  |  |  |  | -1.71 | 0.0003 |  |  |
|  | NTHI1292111200001125.5 |  |  |  |  | -1.74 | 0.0003 |  |  |
|  | NTHI1292111200001125.7 |  |  |  |  | -1.74 | 0.0006 |  |  |
|  | NTHI1292111200001125.4 |  |  |  |  | -1.80 | 0.0002 |  |  |
|  | NTHI1292111200001125.2 |  |  |  |  | -1.81 | 0.0001 | 0.0003 |  |
|  |  |  |  |  |  | *Mean -1.74* |  |  |  |
|  | NTHI0477111200000412.6 | NTHI0477 | Q4QNI3 | hfeD | ABC-type chelated iron transport system | -1.50099 | 0.0014 | 0.001 |  |
|  |  |  |  |  |  |  |  |  |  |
|  |  |  |  |  |  |  |  |  |  |
| **DNA packaging** | NTHI1741111200001526.7 | NTHI1741 | Q4QKB9 |  | phage terminase large subunit | -1.99 | 0.0014 |  |  |
|  | NTHI1741111200001526.5 |  |  |  |  | -2.01 | 0.0013 |  |  |
|  | NTHI1741111200001526.1 |  |  |  |  | -2.05 | 0.0011 | 0.001 |  |
|  |  |  |  |  |  | *Mean -2.02* |  |  |  |
|  |  |  |  |  |  |  |  |  |  |
| **Protein deacetylation** | NTHI1634111200001430.1 | NTHI1634 | Q4QKL5 |  | SIR2 family | -2.73 | 0.0001 |  |  |
|  | NTHI1634111200001430.3 |  |  |  |  | -2.74 | 0.0001 |  |  |
|  | NTHI1634111200001430.7 |  |  |  |  | -2.77 | 0.0002 |  |  |
|  | NTHI1634111200001430.6 |  |  |  |  | -2.77 | 0.0002 |  |  |
|  | NTHI1634111200001430.5 |  |  |  |  | -2.81 | 0.0002 |  |  |
|  | NTHI1634111200001430.4 |  |  |  |  | -2.85 | 0.0001 |  |  |
|  | NTHI1634111200001430.2 |  |  |  |  | -2.95 | 0.0001 | 0.0002 |  |
|  |  |  |  |  |  | *Mean -2.80* |  |  |  |
|  |  |  |  |  |  |  |  |  |  |
| **RNA repair** | NTHI1436111200001254.3 | NTHI1436 | Q4QL41 | cca | tRNA adenylyltransferase | -1.97 | 0.0017 | 0.002 |  |
|  |  |  |  |  |  |  |  |  |  |
|  |  |  |  |  |  |  |  |  |  |
| **Amino acid**  **transporter** | NTHI0396111200000341.2 | NTHI0396 | Q4QNQ4 | mtr | tryptophan permease | -1.76 | 0.0001 |  |  |
|  | NTHI0396111200000341.7 |  |  |  |  | -1.78 | 0.0005 |  |  |
|  | NTHI0396111200000341.4 |  |  |  |  | -1.83 | 0.0002 |  |  |
|  | NTHI0396111200000341.3 |  |  |  |  | -1.85 | 0.0002 |  |  |
|  | NTHI0396111200000341.6 |  |  |  |  | -1.86 | 0.0003 |  |  |
|  | NTHI0396111200000341.1 |  |  |  |  | -1.89 | 0.0000 |  |  |
|  | NTHI0396111200000341.5 |  |  |  |  | -1.92 | 0.0002 | 0.0002 |  |
|  |  |  |  |  |  | *Mean -1.84* |  |  |  |
|  |  |  |  |  |  |  |  |  |  |
| **Unknown** | NTHI0215111200000189.3 | NTHI0215 | Q4QP56 |  | hypothetical protein | -3.17 | 0.0006 |  | transmembrane transport |
|  | NTHI0215111200000189.5 |  |  |  |  | -3.21 | 0.0009 |  |  |
|  | NTHI0215111200000189.4 |  |  |  |  | -3.26 | 0.0008 |  |  |
|  | NTHI0215111200000189.7 |  |  |  |  | -3.27 | 0.0011 |  |  |
|  | NTHI0215111200000189.6 |  |  |  |  | -3.34 | 0.0011 |  |  |
|  | NTHI0215111200000189.2 |  |  |  |  | -3.34 | 0.0004 |  |  |
|  | NTHI0215111200000189.1 |  |  |  |  | -3.35 | 0.0003 | 0.001 |  |
|  |  |  |  |  |  | *Mean -3.28* |  |  |  |
|  | NTHI0680111200000599.7 | NTHI0680 | Q4QMZ6 |  | hypothetical protein | -1.70 | 0.0009 |  |  |
|  | NTHI0680111200000599.6 |  |  |  |  | -1.71 | 0.0007 |  |  |
|  | NTHI0680111200000599.1 |  |  |  |  | -1.71 | 0.0002 |  |  |
|  | NTHI0680111200000599.4 |  |  |  |  | -1.72 | 0.0002 |  |  |
|  | NTHI0680111200000599.3 |  |  |  |  | -1.73 | 0.0002 |  |  |
|  | NTHI0680111200000599.5 |  |  |  |  | -1.74 | 0.0003 |  |  |
|  | NTHI0680111200000599.2 |  |  |  |  | -1.78 | 0.0002 | 0.0004 |  |
|  |  |  |  |  |  | *Mean -1.73* |  |  |  |
|  | NTHI0735111200000649.5 | NTHI0735 | Q4QMU6 |  | hypothetical protein | -1.56 | 0.0007 |  | Predicted membrane protein |
|  | NTHI0735111200000649.2 |  |  |  |  | -1.58 | 0.0002 |  |  |
|  | NTHI0735111200000649.6 |  |  |  |  | -1.58 | 0.0007 |  |  |
|  | NTHI0735111200000649.3 |  |  |  |  | -1.63 | 0.0002 |  |  |
|  | NTHI0735111200000649.4 |  |  |  |  | -1.65 | 0.0004 |  |  |
|  | NTHI0735111200000649.7 |  |  |  |  | -1.67 | 0.0013 |  |  |
|  | NTHI0735111200000649.1 |  |  |  |  | -1.80 | 0.0001 | 0.0005 |  |
|  |  |  |  |  |  | *Mean -1.64* |  |  |  |
|  | NTHI1330111200001160.4 | NTHI1330 | Q4QLD5 |  | hypothetical protein | -1.61 | 0.0016 |  |  |
|  | NTHI1330111200001160.6 |  |  |  |  | -1.63 | 0.0016 |  |  |
|  | NTHI1330111200001160.1 |  |  |  |  | -1.65 | 0.0007 |  |  |
|  | NTHI1330111200001160.3 |  |  |  |  | -1.65 | 0.0010 |  |  |
|  | NTHI1330111200001160.2 |  |  |  |  | -1.66 | 0.0008 | 0.001 |  |
|  |  |  |  |  |  | *Mean -1.64* |  |  |  |
|  | NTHI1331111200001161.2 | NTHI1331 | Q4QLD4 |  | hypothetical protein | -1.71 | 0.0012 |  | Dehydrogenase |
|  | NTHI1331111200001161.1 |  |  |  |  | -1.75 | 0.0010 |  |  |
|  | NTHI1331111200001161.4 |  |  |  |  | -1.76 | 0.0013 |  |  |
|  | NTHI1331111200001161.6 |  |  |  |  | -1.79 | 0.0013 |  |  |
|  | NTHI1331111200001161.7 |  |  |  |  | -1.85 | 0.0014 | 0.001 |  |
|  |  |  |  |  |  | *Mean -1.77* |  |  |  |
|  | NTHI1505111200001314.1 | NTHI1505 | Q4QKY1 |  | hypothetical protein | -2.18 | 0.0007 |  |  |
|  | NTHI1505111200001314.5 |  |  |  |  | -2.21 | 0.0012 |  |  |
|  | NTHI1505111200001314.3 |  |  |  |  | -2.24 | 0.0010 |  |  |
|  | NTHI1505111200001314.2 |  |  |  |  | -2.24 | 0.0009 |  |  |
|  | NTHI1505111200001314.4 |  |  |  |  | -2.25 | 0.0012 |  |  |
|  | NTHI1505111200001314.6 |  |  |  |  | -2.26 | 0.0014 |  |  |
|  | NTHI1505111200001314.7 |  |  |  |  | -2.34 | 0.0014 | 0.001 |  |
|  |  |  |  |  |  | *Mean -2.25* |  |  |  |
|  | NTHI1511111200001319.7 | NTHI1511 | Q4QKX6 |  | hypothetical protein | -2.39 | 0.0010 |  | DNA replication |
|  | NTHI1511111200001319.4 |  |  |  |  | -2.47 | 0.0007 |  |  |
|  | NTHI1511111200001319.5 |  |  |  |  | -2.51 | 0.0007 |  |  |
|  | NTHI1511111200001319.3 |  |  |  |  | -2.55 | 0.0007 |  |  |
|  | NTHI1511111200001319.2 |  |  |  |  | -2.59 | 0.0006 |  |  |
|  | NTHI1511111200001319.6 |  |  |  |  | -2.62 | 0.0008 |  |  |
|  | NTHI1511111200001319.1 |  |  |  |  | -2.62 | 0.0004 | 0.0007 |  |
|  |  |  |  |  |  | *Mean -2.54* |  |  |  |
|  | NTHI1528111200001334.7 | NTHI1528 | Q4QKW1 |  | hypothetical protein | -2.13 | 0.0008 |  |  |
|  | NTHI1528111200001334.2 |  |  |  |  | -2.14 | 0.0003 |  |  |
|  | NTHI1528111200001334.6 |  |  |  |  | -2.16 | 0.0008 |  |  |
|  | NTHI1528111200001334.3 |  |  |  |  | -2.16 | 0.0004 |  |  |
|  | NTHI1528111200001334.1 |  |  |  |  | -2.16 | 0.0002 |  |  |
|  | NTHI1528111200001334.4 |  |  |  |  | -2.17 | 0.0004 |  |  |
|  | NTHI1528111200001334.5 |  |  |  |  | -2.24 | 0.0005 | 0.0005 |  |
|  |  |  |  |  |  | *Mean -2.17* |  |  |  |
|  | NTHI1534111200001340.3 | NTHI1534 | Q4QKV5 |  | hypothetical protein | -1.84 | 0.0015 |  |  |
|  | NTHI1534111200001340.4 |  |  |  |  | -1.84 | 0.0016 | 0.002 |  |
|  |  |  |  |  |  | *Mean -1.84* |  |  |  |
|  | NTHI1635111200001431.2 | NTHI1635 | Q4QKL4 |  | hypothetical protein | -3.75 | 0.0010 |  | catalytic activity |
|  | NTHI1635111200001431.7 |  |  |  |  | -4.02 | 0.0017 |  |  |
|  | NTHI1635111200001431.6 |  |  |  |  | -4.02 | 0.0017 |  |  |
|  | NTHI1635111200001431.5 |  |  |  |  | -4.05 | 0.0016 |  |  |
|  | NTHI1635111200001431.4 |  |  |  |  | -4.06 | 0.0014 |  |  |
|  | NTHI1635111200001431.1 |  |  |  |  | -4.21 | 0.0009 |  |  |
|  | NTHI1635111200001431.3 |  |  |  |  | -4.44 | 0.0013 | 0.001 |  |
|  |  |  |  |  |  | *Mean -4.08* |  |  |  |
|  | NTHI1721111200001507.1 | NTHI1721 | Q4QKD8 |  | hypothetical protein | -3.02 | 0.0008 |  | DNA binding |
|  | NTHI1721111200001507.6 |  |  |  |  | -3.05 | 0.0010 |  |  |
|  | NTHI1721111200001507.2 |  |  |  |  | -3.08 | 0.0010 |  |  |
|  | NTHI1721111200001507.7 |  |  |  |  | -3.10 | 0.0015 |  |  |
|  | NTHI1721111200001507.5 |  |  |  |  | -3.16 | 0.0010 |  |  |
|  | NTHI1721111200001507.4 |  |  |  |  | -3.17 | 0.0010 |  |  |
|  | NTHI1721111200001507.3 |  |  |  |  | -3.18 | 0.0010 | 0.001 |  |
|  |  |  |  |  |  | *Mean -3.11* |  |  |  |
|  | NTHI1726111200001512.7 | NTHI1726 | Q4QKD3 |  | hypothetical protein | -1.88 | 0.0008 |  |  |
|  | NTHI1726111200001512.6 |  |  |  |  | -1.88 | 0.0007 |  |  |
|  | NTHI1726111200001512.2 |  |  |  |  | -1.90 | 0.0006 |  |  |
|  | NTHI1726111200001512.5 |  |  |  |  | -1.94 | 0.0006 |  |  |
|  | NTHI1726111200001512.1 |  |  |  |  | -1.95 | 0.0004 |  |  |
|  | NTHI1726111200001512.3 |  |  |  |  | -1.95 | 0.0006 |  |  |
|  | NTHI1726111200001512.4 |  |  |  |  | -1.95 | 0.0006 | 0.0006 |  |
|  |  |  |  |  |  | *Mean -1.92* |  |  |  |
|  | NTHI1735111200001520.2 | NTHI1735 | Q4QKC5 |  | hypothetical protein | -2.26 | 0.0009 |  |  |
|  | NTHI1735111200001520.5 |  |  |  |  | -2.33 | 0.0012 |  |  |
|  | NTHI1735111200001520.6 |  |  |  |  | -2.33 | 0.0017 |  |  |
|  | NTHI1735111200001520.4 |  |  |  |  | -2.33 | 0.0012 | 0.001 |  |
|  |  |  |  |  |  | *Mean -2.31* |  |  |  |
|  | NTHI1736111200001521.5 | NTHI1736 | Q4QKC4 |  | hypothetical protein | -2.06 | 0.0009 |  | Cell wall catabolism |
|  | NTHI1736111200001521.7 |  |  |  |  | -2.08 | 0.0010 |  |  |
|  | NTHI1736111200001521.1 |  |  |  |  | -2.08 | 0.0005 |  |  |
|  | NTHI1736111200001521.4 |  |  |  |  | -2.10 | 0.0008 |  |  |
|  | NTHI1736111200001521.6 |  |  |  |  | -2.11 | 0.0010 |  |  |
|  | NTHI1736111200001521.2 |  |  |  |  | -2.12 | 0.0005 |  |  |
|  | NTHI1736111200001521.3 |  |  |  |  | -2.15 | 0.0008 | 0.0008 |  |
|  |  |  |  |  |  | *Mean -2.10* |  |  |  |
|  | NTHI1738111200001523.6 | NTHI1738 | Q4QKC2 |  | hypothetical protein | -1.99 | 0.0004 |  |  |
|  | NTHI1738111200001523.4 |  |  |  |  | -1.99 | 0.0003 |  |  |
|  | NTHI1738111200001523.7 |  |  |  |  | -2.03 | 0.0005 |  |  |
|  | NTHI1738111200001523.3 |  |  |  |  | -2.05 | 0.0003 |  |  |
|  | NTHI1738111200001523.5 |  |  |  |  | -2.06 | 0.0004 |  |  |
|  | NTHI1738111200001523.1 |  |  |  |  | -2.09 | 0.0002 |  |  |
|  | NTHI1738111200001523.2 |  |  |  |  | -2.10 | 0.0003 | 0.0003 |  |
|  |  |  |  |  |  | *Mean -2.04* |  |  |  |
|  | NTHI1739111200001524.5 | NTHI1739 | Q4QKC1 |  | hypothetical protein | -2.09 | 0.0004 |  | DNA binding |
|  | NTHI1739111200001524.6 |  |  |  |  | -2.11 | 0.0008 |  |  |
|  | NTHI1739111200001524.1 |  |  |  |  | -2.11 | 0.0003 |  |  |
|  | NTHI1739111200001524.7 |  |  |  |  | -2.12 | 0.0008 |  |  |
|  | NTHI1739111200001524.4 |  |  |  |  | -2.13 | 0.0004 |  |  |
|  | NTHI1739111200001524.3 |  |  |  |  | -2.14 | 0.0004 |  |  |
|  | NTHI1739111200001524.2 |  |  |  |  | -2.15 | 0.0003 | 0.0005 |  |
|  |  |  |  |  |  | *Mean -2.12* |  |  |  |
|  | NTHI1727111200001513.7 | NTHI1727 | Q4QKD2 | ninB | recombination protein NinB | -2.48 | 0.0008 |  |  |
|  | NTHI1727111200001513.6 |  |  |  |  | -2.49 | 0.0007 |  |  |
|  | NTHI1727111200001513.1 |  |  |  |  | -2.51 | 0.0004 |  |  |
|  | NTHI1727111200001513.3 |  |  |  |  | -2.52 | 0.0005 |  |  |
|  | NTHI1727111200001513.5 |  |  |  |  | -2.54 | 0.0006 |  |  |
|  | NTHI1727111200001513.4 |  |  |  |  | -2.61 | 0.0005 |  |  |
|  | NTHI1727111200001513.2 |  |  |  |  | -2.61 | 0.0004 | 0.0006 |  |
|  |  |  |  |  |  | *Mean -2.54* |  |  |  |
|  | NTHI1728_1111200001514.7 | NTHI1728 | Q4QKD1 | ninG | recombination protein NinG | -2.42 | 0.0010 |  |  |
|  | NTHI1728_1111200001514.3 |  |  |  |  | -2.46 | 0.0006 |  |  |
|  | NTHI1728_1111200001514.6 |  |  |  |  | -2.47 | 0.0007 |  |  |
|  | NTHI1728_1111200001514.4 |  |  |  |  | -2.48 | 0.0006 |  |  |
|  | NTHI1728_1111200001514.5 |  |  |  |  | -2.52 | 0.0007 |  |  |
|  | NTHI1728_1111200001514.2 |  |  |  |  | -2.56 | 0.0005 |  |  |
|  | NTHI1728_1111200001514.1 |  |  |  |  | -2.53 | 0.0004 | 0.0007 |  |
|  |  |  |  |  |  | *Mean -2.49* |  |  |  |
